# The genome of the pygmy right whale illuminates the evolution of rorquals

**DOI:** 10.1186/s12915-023-01579-1

**Published:** 2023-04-12

**Authors:** Magnus Wolf, Konstantin Zapf, Deepak Kumar Gupta, Michael Hiller, Úlfur Árnason, Axel Janke

**Affiliations:** 1 Senckenberg Biodiversity and Climate Research Centre (BiK-F), Georg-Voigt-Strasse 14-16, Frankfurt Am Main, Germany; 2 Institute for Ecology, Evolution and Diversity, Goethe University, Max-Von-Laue-Strasse. 9, Frankfurt Am Main, Germany; 3 LOEWE-Centre for Translational Biodiversity Genomics (TBG), Senckenberg Nature Research Society, Georg-Voigt-Straße 14-16, Frankfurt Am Main, Germany; 4 Institute of Cell Biology and Neuroscience, Goethe University Frankfurt, Max-Von-Laue-Str. 9, Frankfurt Am Main, Germany; 5 Department of Clinical Sciences Lund, Lund University, Lund, Sweden; 6 Department of Neurosurgery, Skane University Hospital in Lund, Lund, Sweden

**Keywords:** Pygmy right whale, *Caperea marginata*, Whole-genome sequencing, Phylogenomics, Rorquals, Positive selection, Cancer resistance, Peto’s paradox

## Abstract

**Background:**

Baleen whales are a clade of gigantic and highly specialized marine mammals. Their genomes have been used to investigate their complex evolutionary history and to decipher the molecular mechanisms that allowed them to reach these dimensions. However, many unanswered questions remain, especially about the early radiation of rorquals and how cancer resistance interplays with their huge number of cells. The pygmy right whale is the smallest and most elusive among the baleen whales. It reaches only a fraction of the body length compared to its relatives and it is the only living member of an otherwise extinct family. This placement makes the pygmy right whale genome an interesting target to update the complex phylogenetic past of baleen whales, because it splits up an otherwise long branch that leads to the radiation of rorquals. Apart from that, genomic data of this species might help to investigate cancer resistance in large whales, since these mechanisms are not as important for the pygmy right whale as in other giant rorquals and right whales.

**Results:**

Here, we present a first de novo genome of the species and test its potential in phylogenomics and cancer research. To do so, we constructed a multi-species coalescent tree from fragments of a whole-genome alignment and quantified the amount of introgression in the early evolution of rorquals. Furthermore, a genome-wide comparison of selection rates between large and small-bodied baleen whales revealed a small set of conserved candidate genes with potential connections to cancer resistance.

**Conclusions:**

Our results suggest that the evolution of rorquals is best described as a hard polytomy with a rapid radiation and high levels of introgression. The lack of shared positive selected genes between different large-bodied whale species supports a previously proposed convergent evolution of gigantism and hence cancer resistance in baleen whales.

**Supplementary Information:**

The online version contains supplementary material available at 10.1186/s12915-023-01579-1.

## Teaser

The genome of the smallest baleen whale was used to update the rorqual phylogeny and to identify genes related to cancer resistance.

## Background

### Biology of *Caperea marginata*

Baleen whales (*Mysticeti*) are the largest animals on earth, reaching up to 30 m in length and a weight of 150 metric tons. These iconic animals have received considerable public and scientific interest in the past. The pygmy right whale (*Caperea marginata*, Gray 1846) is the smallest species among the baleen whales, with records ranging between 5 and 6.5 m in length and weighing 3t to 3.5t [1]. They have a circumpolar distribution around the southern hemisphere, although crossing equatorial regions and hence a wider distribution may be possible [2]. The biology of this species is still poorly understood, not only because the number of sightings is very limited, but also because of possible confusions with minke whales (*Balaenoptera acutorostrata* and *B. bonaerensis*) [1]. They are believed to be slow swimming filter feeders given the morphology of their feeding apparatus that is similar to that of the right whales (*Balaenidae*) [3, 4] and it is assumed that they are not deep divers because their heart and lungs are, compared to such whales, relatively small [5]. There is no information available about their abundance, but they were not targeted by whalers in the past [1]. The species is listed as “least concern” on the IUCN red list due to the lack of information [6].

### Phylogeny of *Caperea marginata*

Multiple features of the skull and skeletal morphology of the pygmy right whale are unique within the baleen whales [7], leading to a complex history of taxonomic re-assignments and re-naming of the species [8]. Many morphological studies suggested that the species is an early diverging member of the right whales [9–11], hence its common name: pygmy right whale. However, molecular studies have consistently reported a closer relationship to the rorquals, *Balaenopteridae* [12–17]. In recent years, the species was allocated closer to the rorquals and placed in the otherwise extinct family of *Cetotheriidae* and the subfamily of *Neobalaenidae* based on a combination of extensive comparisons to fossil records and on molecular data [3]. This placement was later supported by a phylogenomic study that included nearly all extant *Cetacea* species [18].

### Phylogeny problems of rorquals

While placing the pygmy right whale in the *Cetotheriidae* and hence the *Neobalaenidae* seems resolved, placing and ordering groups within the *Balaenopteridae*, rorquals, remains challenging. It is assumed that the rorquals experienced a rapid radiation in combination with incomplete lineage sorting (ILS) and introgression after diverging from a common ancestor 10 to 25 million years ago [14, 18–20]. Especially the phylogenetic position of the gray whale (*Balaenoptera robustus*, formerly *Eschrichtius robustus*, see Årnason et al. (2018) [20]) remains uncertain because even recent phylogenomic analyses addressing this clade resulted in short branches with low support despite their plethora of molecular data [18, 20]. However, current studies were either limited by taxon sampling due to the lack of whole-genome sequences [20] or might have been hampered by the limited amount of evolutionary information per short protein-coding sequences [18]. Revisiting the problematic phylogeny of rorquals with an increased taxon-sampling and long whole-genome alignment (WGA) fragments rather than short coding sequences is expected to increase the resolution of this rapidly diverged species complex. The genome of the pygmy right whale will most likely improve the resolution of rorqual evolution because of its placement at the base of the rorqual divergence and its addition to phylogenomic analyses will split up an otherwise long branch separating rorquals from right whales.

### Peto’s paradox and cancer research

Baleen whales have also received substantial interest in research regarding cancer resistance because of their relatively normal cancer mortality despite their large number of cells and relatively long life-expectancy known as the “Peto’s” paradox [21–24]. In previous attempts, the identification of related oncogenes and tumor suppressor genes (TSG) was often based on a genome-wide comparison of selection rates or gene copy numbers between a large species and a smaller relative [22, 24, 25]. Although these pairwise comparisons resulted in numerous candidate genes that may be responsible for the resistance to cancer in baleen whales, their identification has remained vague because previous studies were restricted to a single species pair, given that only the minke whale genome [26] was available as a small-bodied reference. Alternative approaches exist, such as codon-based models that estimate selective pressure along evolutionary branches [22, 24], but they need to be treated with caution because model misspecification and alignment errors can result in a potentially high number of artifacts [27]. Increasing the number of pairs available for selection rate comparisons could dramatically increase their precision. Therefore, adding a reference genome for the considerably smaller pygmy right whale will be a valuable addition to this kind of research and will likely reduce the risk of identifying false positive candidate genes.

### Objectives

In this study, we assemble a de novo reference genome for the pygmy right whale (*Caperea marginata,* Gray 1846) and test its potential to improve the phylogenetic resolution among the rorquals and to increase the chance to identify potential cancer-related genes. Therefore, we include the new genome in a reissued phylogenomic analysis with an increased sampling of whole-genome sequences and long WGA fragments rather than short coding sequences. A set of candidate genes related to cancer resistance is compiled by comparing selection rates between several large-bodied baleen whales and small-bodied relatives. Additionally, we provide a first estimate of the genetic diversity and model the demographic history of the species to provide new insights into this elusive species.

## Results

### Genome characteristics

The genome of the pygmy right whale was assembled to a total size of 2.5 Gbp and consists of 51,945 contigs with an N50 of 112.3 kbp and an L50 of 6438 contigs (Table 1). The GC content of the finale assembly is 41.2%, scaffolds contain 8920.3 N’s per 100 kbp and the genome-wide heterozygosity is 0.11%. BUSCO completeness analyses of the three OrthoDB clades *Cetartiodactyla*, *Laurasiatheria*, and *Mammalia* yielded 63.7%, 66.7%, and 65.7% complete core genes, respectively, as well as 9.9%, 14.3%, and 14.1% fragmented genes. A de novo modeling and masking of repeats found that 37.8% of the genome is covered by interspersed repeats (Additional file 1: Table S1). Homology-based annotation yielded 33,644 potential transcripts, of which 95.7% were functionally annotated.

**Table 1:** Summary statistics, BUSCO completeness analysis, and annotation statistics for the *C. marginate* reference genome

**Assembly statistics**	
**No. contigs**	**51,950**
**No. contigs (> 50 kbp)**	**16,071**
**L50**	**9883**
**N50 (bp)**	**112,264**
**Total length (bp)**	**2,515,163,484**
**GC (%)**	**41.19**
**No. of *N*s per 100 kb**	**8920.26**
**Heterozygosity (%)**	**0.11**
**BUSCO completeness**	
**BUSCO (cetartiodactyla)**	**C: 63.7%[S: 61.9%, D:1.8%]**
	**F: 9.9%, M: 26.4%**
	***n*: 13,335**
**BUSCO (laurasiatheria)**	**C: 66.7%[S: 64.7%, D: 2.0%]**
	**F: 14.3%, M: 19.0%**
	***n*: 12,234**
**BUSCO (mammalia)**	**C: 65.6%[S: 63.6%, D: 2.0%]**
	**F: 14.1%, M: 20.3%**
	***n*: 9226**
**Annotation statistics**	
**Total interspersed repeats (bp)**	**951,069,063 (37.81%)**
**Number of transcripts**	**32,808**
**Functional annotated genes**	**28,267 (86.1%)**

*BUSCO* Benchmarking Universal Single Copy Orthologs, *C* Complete, *S* Single copy, *D* Duplicated, *F* fragmented, *M* Missing

### Phylogenomics

**Fig. 1:**
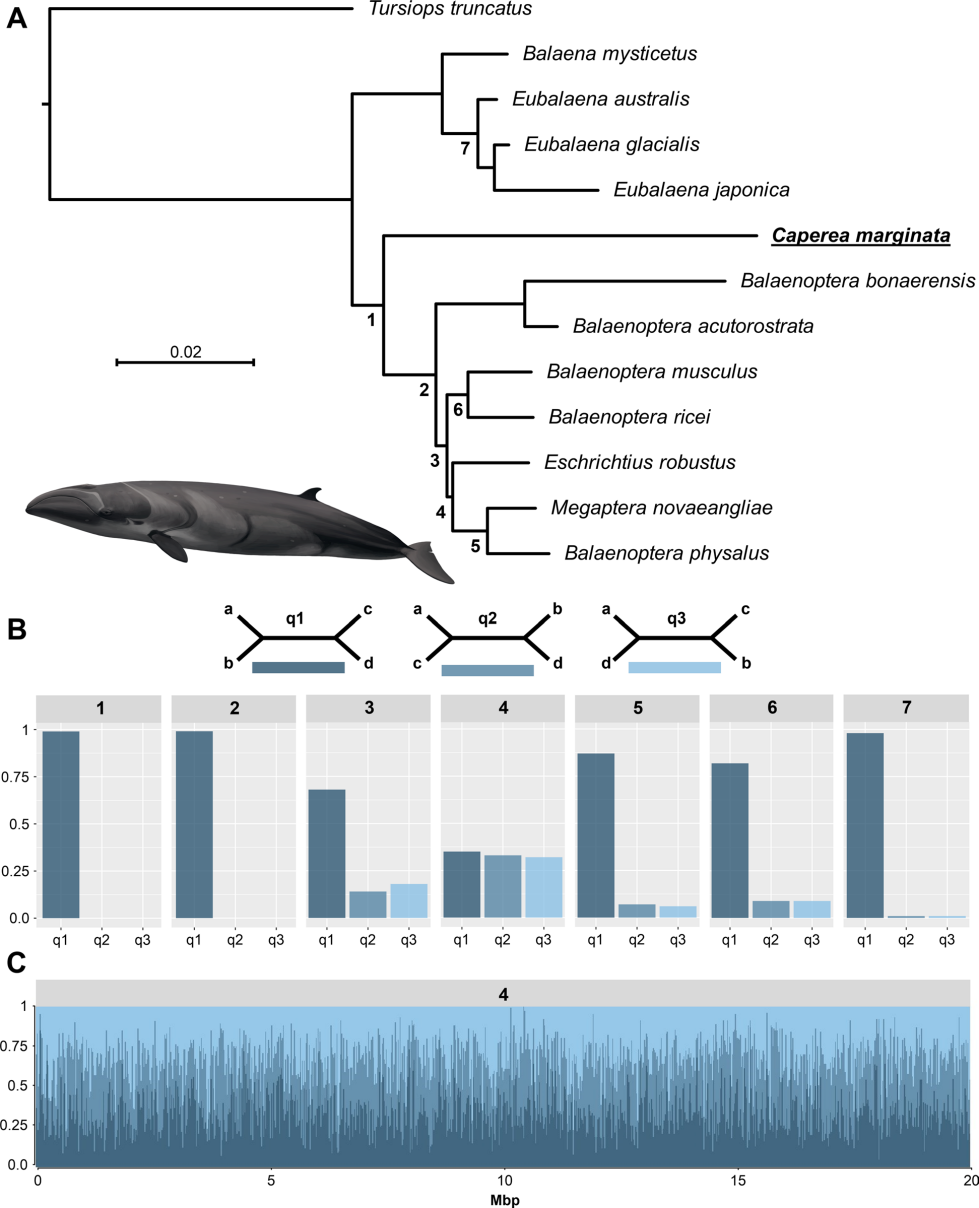
Phylogenomic analysis of baleen whales using whole-genome alignment fragments. **A** Phylogenomic multi-species coalescent (MSC) tree inferred from 46,941 trees that were each constructed from a 20 kbp whole-genome alignment fragment. All branches conceived 1.0 local posterior probabilities and branch lengths were added by a maximum likelihood inference using amino acid sequences of 563 high-quality single copy ortholog sequences. The pygmy right whale was placed at the base of the rorquals, and the gray whale was grouped together with the humpback whale and fin whale. **B** Quartet scores of different branches across the MSC tree. Branches 1– 7 were analyzed for the number of trees supporting one of the three possible unrooted topologies (q1– q3). Branch 4, representing the position of the gray whale, received nearly equal quartet scores for all alternative topologies. **C** Distribution of quartet scores across the first 20 Mbp of chromosome one of the reference assembly (*Tursiops truncatus*), given three different topologies of branch 4 (light to dark blue). Across the chromosome, no clear runs of shared phylogenetic signals could be identified. Pygmy right whale illustration made by Frédérique Lucas. The assembly data used to generate the results shown can be found in Additional file 1: Table S6 [28–38]

A multispecies coalescent (MSC) phylogenomic tree (Fig. 1A) was constructed for the entire *Mysticeti* including whole-genomes from twelve extant baleen whale species. The tree was conflated from 46,941 individual maximum likelihood (ML) trees that were each constructed from 20 kbp fragments cut from an 1.3 Gbp long whole-genome alignment (WGA) using the genome of the bottlenose dolphin (*Tursiops truncatus*) as a reference. Each fragment contains a mean of ~ 730 parsimony informative sites and the ideal fragment size was determined to be 20 kbp using an approximately unbiased test (Additional file 1: Fig. S1). All branches of the final tree are supported with local posterior probabilities of 1.0 and final branch lengths were estimated using a maximum likelihood inference based on 563 high-quality shared single-copy orthologous amino acid sequences (SCOS). The resulting tree depicts a clear separation between the three baleen whale families: *Balaenidae, Balaenopteridae*, and *Cetotheriidae.* Within the rorquals, the tree supports a grouping of the gray whale with the pair of fin whale and humpback whale, while a sister clade is formed by the blue whale and the rice whale.

For most branches, we found low support for alternative topologies, represented by quartet scores that describe the conflicts between the three possible topologies for an internal, unrooted branch (Fig. 1B). The placement of the gray whale received nearly identical support for all three topologies, with only a slight excess towards the topology presented in the MSC tree. To depict the distribution of these conflicts across the genome, we calculated quartet scores for every 20 kbp fragment of the largest reference chromosome 1, given the overall MSC tree. This analysis revealed an even distribution of signals between the three possible topologies (Fig. 1C) over the entire chromosome with no clear runs of shared phylogenetic signals.

**Fig. 2:**
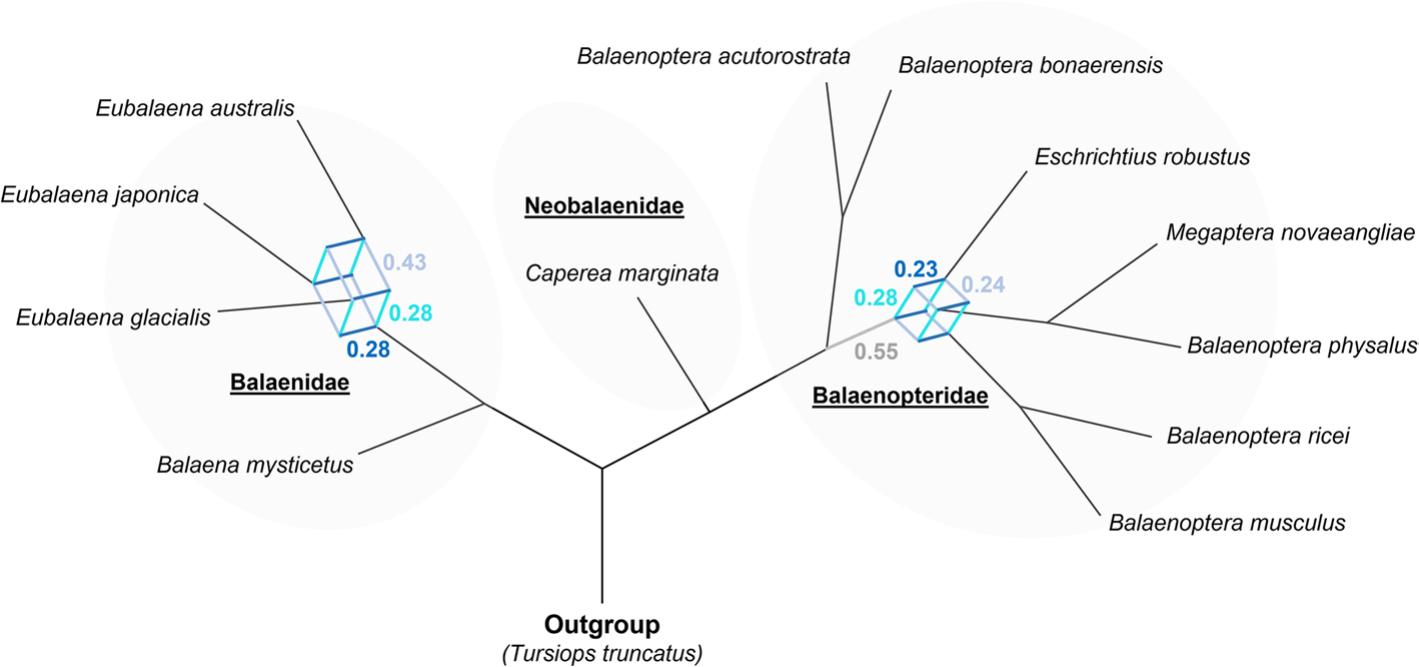
Consensus network of baleen whale evolution based on whole-genome alignment fragments. The network was constructed from 46,941 fragments of 20 kbp length and a 12% threshold was used to depict conflicts. Extensive phylogenetic conflicts characterize the placement of the gray whale consistent with branch 4 of the main phylogenomic analysis

The conflicting phylogenetic signals are also evident within a consensus network constructed from the entire set of 46,941 WGA fragments (Fig. 2). Using a threshold of 12% conflicting edges, we received conflicting topologies at the base of the *Balaenidae, Balaenopteridae*, and for the placement of the gray whale with the latter having the most even distribution of conflicts. Lower thresholds resulted in more complex patterns indicating additional, though less frequent, conflicting phylogenetic signals from individual WGA fragments (Additional file 1: Fig. S2).

To evaluate whether these conflicting signals originated from ILS or introgression, we quantified introgression events via branch lengths (QuIBL) as presented by Edelman et al. 2019 [39]. Based on the distribution of internal branch lengths of discordant triplet topologies, the test determines whether conflicting trees were the result from either ILS only (H_0_), or ILS together with introgression (H_1_). In the case of ILS only (H_0_), branch lengths are expected to be exponentially distributed. Restricting ourselves to all rorquals in close evolutionary proximity to the contested gray whale, we found evidence for introgression (ΔBIC <  − 10) in 5 out of 10 possible triplets (Additional file 1: Table S2). We found on average 33% discordant WGA fragment trees per triplet of which ~ 58% were likely the result of past introgression events. Thus, of the total number of evaluated trees, a mean of 19% (H_1_) is estimated to be affected by introgression while the other 14% showed an exponential distribution of internal branch lengths and are therefore considered to be the result of ILS only (H_0_). Triplets that included the gray whale as well as one representative of both possible sister clades usually showed around 66% discordant trees of which 64%, or 42% of the total data set, likely had a history of introgression. The triplet of gray whale, fin whale, and blue whale resulted in the most signals of introgression with 48.1% of all tested trees. Hence, we assume introgression to be the dominant driver for conflicting trees in our dataset, especially around the contested position of the gray whale.

Additionally, we constructed an MSC tree from 563 maximum likelihood inferences of single-copy orthologous sequences (SCOS) (Additional file 1: Fig. S3) and an MSC tree directly from 1.7 million single nucleotide polymorphisms (SNPs), called after mapping available short-read data from nine baleen whale species to the bowhead whale (*Balaena mysticetus*) reference genome (Additional file 1: Fig. S4, [22]. Both approaches resulted in different placements of the gray whale with either low bootstrap support values or higher amounts of quartet score conflicts. The consensus phylogeny inferred from SCOS gene trees placed the gray whale at the base of the (fin whale, humpback whale) and (blue whale, rice whale) clades, whereas the MSC phylogeny based on SNPs placed the gray whale together with the blue and rice whale as a sister clade to the fin whale and humpback whale. A consensus network constructed from SCOS data resulted in inconclusive resolution as well (Additional file 1: Fig. S5). Accordingly, we chose the WGA-based topology for all downstream analyses as it depicts the most parsimonious hypothesis with the fewest conflicts in baleen whales.

**Fig. 3:**
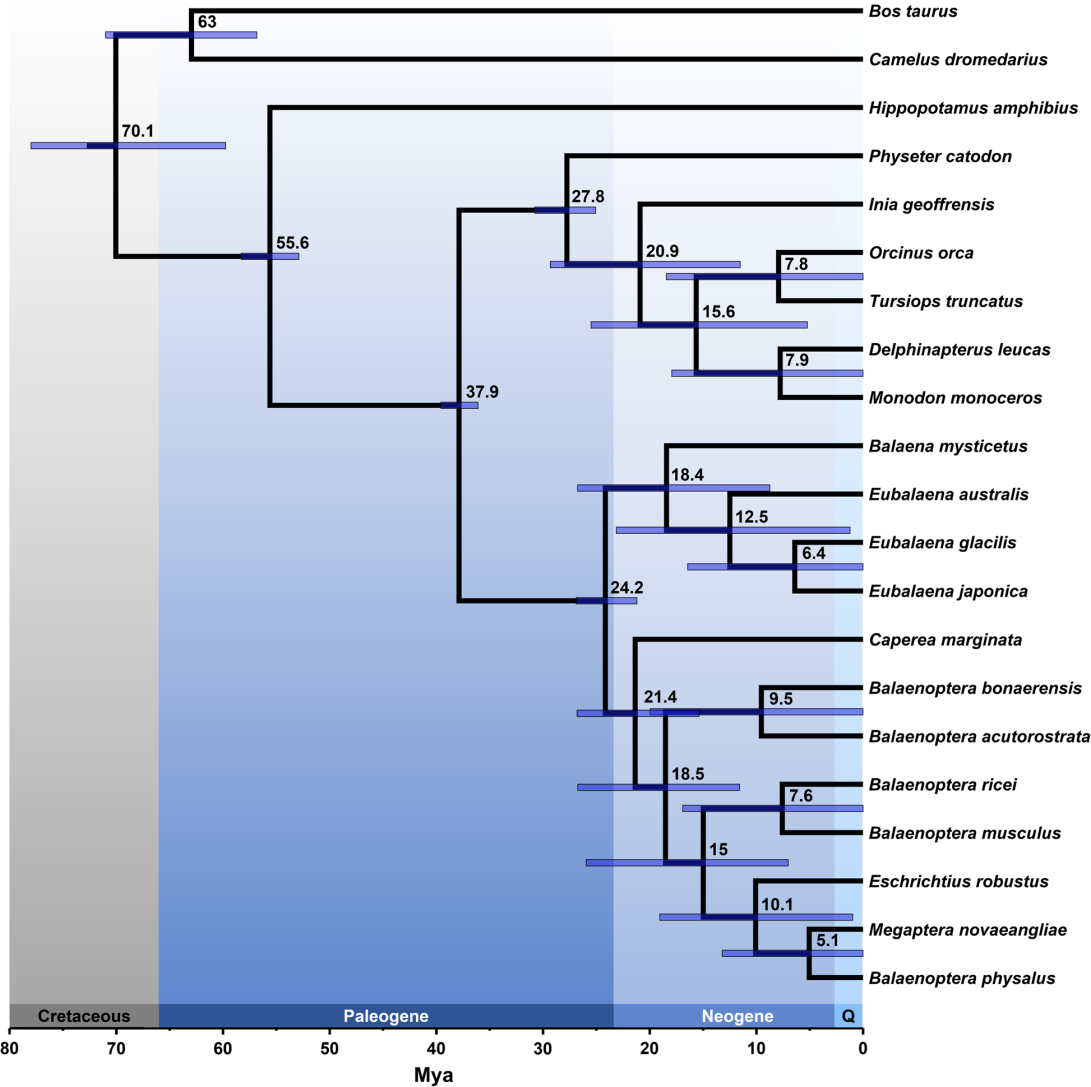
Divergence time estimates of whales (*Cetacea*). The tree was constructed using the topology presented in Fig. 1, five calibration points (Additional file 1: Table S3 [40–44]), and amino acid sequences of 562 single-copy orthologous sequences. According to this estimate, baleen whales originated at 24.2 Mya, the pygmy right whale diverged around 21.4 Mya, and rorquals around 18.5 Mya, although the error bars indicate large ranges for all three cases

Divergence time estimates of baleen whales were estimated based on the topology of the consensus WGA tree using branch lengths from an ML analysis of SCOS amino acid data and five calibration points (Fig. 3, Additional file 1: Table S3 [40–44]). According to these estimates, baleen whales diverged between 26.9 and 21.2 million years ago (Mya), and the split between *Cetotheriidae* and *Balaenopteridae* was estimated to have occurred between 26.8 and 15.4 Mya. Divergence estimated within the rorquals showed a wide range of estimates between 25.9 and 7 Mya.

### Demographic inference

**Fig. 4:**
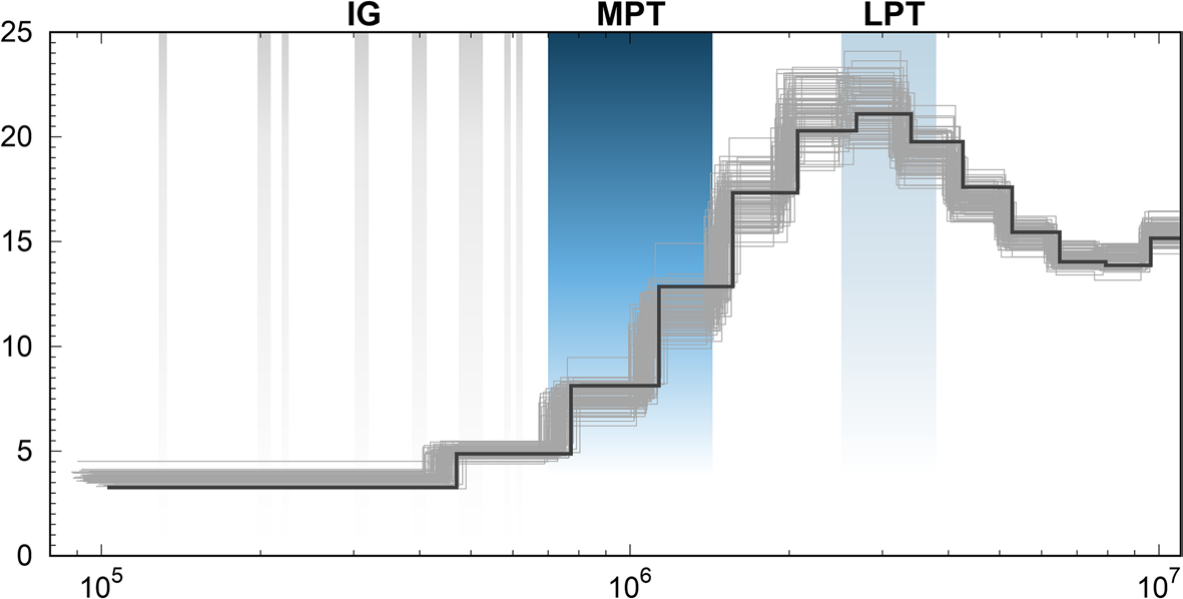
Demographic history of the pygmy right whale inferred using the PSMC framework. The model covers the last 10 Mya to 100 kya and is scaled based on a mutation rate of 1.38 × 10^−8^ per site per generation [20] and a generation time of 22.1 years, using the minke whale as an approximation for the unknown life expectancy [34]. *x*-axis depicts the time in number of years ago while the *y*-axis depicts the effective population size in thousand individuals. The model indicates a peak of effective population size around the Late-Pleistocene Transition (LPT, 2.6 Mya) (light blue), followed by a steady decline until reaching a lower, but stable population size after the Mid-Pleistocene Transition (MPT, 1 Mya–700 kya) (dark blue). Interglacial periods (gray) did not influence stock sizes

The history of the effective population size (Ne) of the pygmy right whale was modeled for a time frame between 10 million years ago (Mya) and 100 thousand years ago (kya) (Fig. 4). The model shows a peak in abundance during the Late-Pleistocene Transition (2.6 Mya) followed by a steady decline until reaching constant numbers after the Mid-Pleistocene Transition (1.6–0.7 Mya) some ~ 400–600 kya. Bootstrap replications closely mirror the initially estimated model, indicating low sampling variance.

### Selection analysis of cancer genes

**Fig. 5:**
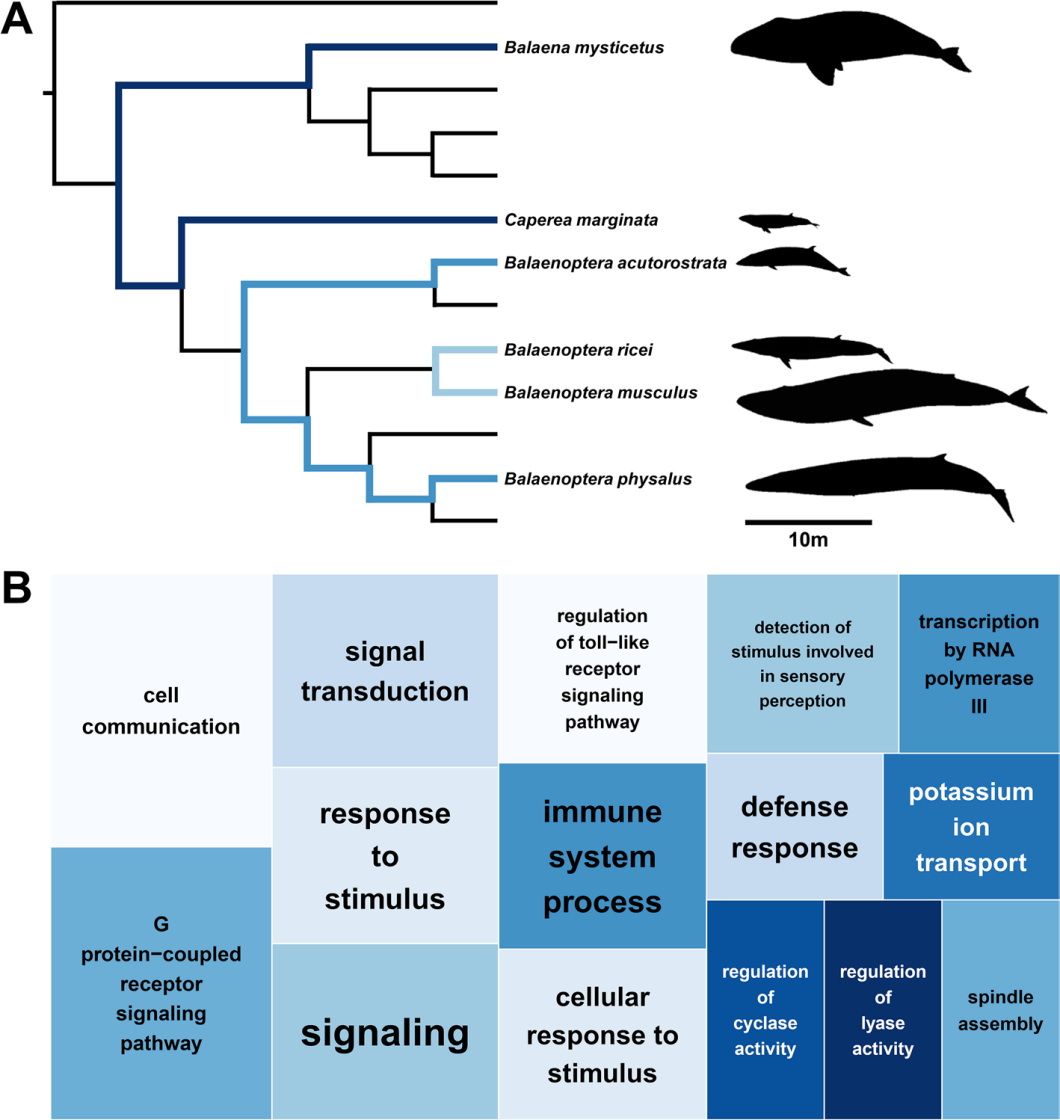
Enriched functions of genes positively selected in large-bodied baleen whales. **A** Pairs of large and small baleen whales identified using phylogenetic targeting [72] based on the species tree presented in Fig. 1 and length and body mass data (Additional file 1: Table S4 [45–65]). Pairs represent the best combination of phylogenetically independent pairs that maximize size differences, putatively related with cancer resistance (Peto’s Paradox, [21]. **B** TreeMap representing gene ontology terms overrepresented in genes with elevated non-synonymous substitution rates in large whales. Rectangle size depicts significance values after false discovery correction after Benjamini and Hochberg

To collect positively selected genes related to body size and hence cancer resistance, we compared rates of non-synonymous (Ka) and synonymous (Ks) substitutions between pairs of large and small-bodied baleen whales. We applied phylogenetic targeting (Additional file 1: Table S4 [45–65], Table S5) and identified three phylogenetically independent pairs that at the same time maximized size differences between them, namely: (1) bowhead whale and pygmy right whale, (2) fin whale and minke whale, and (3) blue whale and rice whale (Fig. 5A). Together with the human reference genome GRCh38, we called single-copy orthologous sequences and collected genes with elevated non-synonymous substitution rates (Ka/Ks > 1) in one or all of the three pairs as a proxy for positive selection. Within our curated set of 1266 single-copy orthologs, we identified 210 unique orthologs with elevated Ka/Ks in at least one of the three pairs. Differentiating between the individual pairs we found: 89 (bowhead/pygmy right), 95 (fin/minke), and 74 (blue/rice) positively selected orthologs, respectively. Six of those genes were found to have an elevated non-synonymous substitution rate in all three pairs (Table 2 [66–70]) that were further functionally specified by BLAST. For five out of six candidate genes, we found evidence in the literature for a correlation between expression patterns and cancer development (Table 2 [66–70]). Furthermore, we found more detailed functional descriptions for two of these genes: first, the C-type lectin domain family 2 member B (CLEC2B/AICL), which is a protein encoded by the natural killer (NK) gene complex proximal CD69 [71]; and second, the RAB15 effector protein (REP15), which together with its associated Rab GTPase controls the flow of transport vesicles in the brain [69]. The ortholog with the highest divergence between Ka and Ks in all three pairs is so far uncharacterized in humans (LOC124907494/LOC124905498) and has only been characterized as “proline-rich” in, e.g., cattle (LOC113892484).

**Table 2:** List of putative cancer-related candidate genes found to be selected (Ka/Ks > 1) in all three pairwise comparisons of large- and small-bodied baleen whales. Genes were identified based on the imbalance between nonsynonymous (Ka) and synonymous (Ks) substitution rates. Pairs were determined using phylogenetic targeting. Sorted by Ka/Ks ratio. Concerning publications: [66–70]

Symbol	HGNC	Orthogroup	Description	Literature	Ka/Ks	*p*-val
*LOC124907494/LOC124905498*	-	OG0018750	Uncharacterized (basic salivary proline-rich protein 1-like in *Bos indicus*)	-	8.19	0.001
C6orf15 (STG)	13,927	OG0018717	Unknown function (expressed in various types of cancer)	Xiong et al., (2022) [66]	2.74	0.165
MGAT4EP	49,418	OG0018311	Pseudogene in human (upregulates the expression of FOXM1 in breast cancer)	Sun et al., (2021) [67]	2.06	0.474
CLEC2B/AICL	2053	OG0018915	Associated with natural killer cells, expressed in various types of cancer	Li et al., (2022) [68]	1.95	0.532
REP15	33,748	OG0018380	Involved in vesicular trafficking, potentially involved in various types of cancer	Rai et al., (2022) [69]	1.67	0.403
C22orf46	26,294	OG0017847	Unknown function (oncogene in adreno-cortical carcinoma)	Li et al., (2020) [70]	1.43	0.435

*HGNC*, HUGO Gene Nomenclature Committee; *HUGO*, Human Genome Organization

We further characterized enriched functions in the set of 210 positively selected genes by performing a gene enrichment analysis of biological processes against the human reference (Fig. 5B, Table 3). We found 20 enriched Gene Ontology (GO) terms most of which represent general terms related to signaling (GO:0,023,052, GO:0,007,165), cell communication (GO:0,007,154), and response to a stimulus (GO:0,050,896, GO:0,051,716). Terms belonging to the G-protein-coupled receptor signaling pathway (GO:0,007,186) were found to be the most significantly enriched with an 8.9-fold enrichment. We also detected GO terms related to immune system processes (GO:0,002,376) and cell defense (GO:0,006,952). Terms occurring only once were excluded from further discussion.

**Table 3:** List GO terms for biological processes overrepresented in genes positively selected (Ka/Ks > 1) in large-bodied baleen whales. GO terms were collected from the human ortholog of genes with elevated non-synonymous substitution rates in large-bodied whales. GO terms from the human genome (GRCh38) were used as reference. Sorted after significance values

Term ID	Description	Number in reference	Number in query	Fold enrichment	*P*-value*
GO:0,007,186	G-protein-coupled receptor signaling pathway	1098	10	8.1	0.0009
GO:0,007,154	Cell communication	4549	14	2.7	0.0009
GO:0,007,165	Signal transduction	4201	12	2.5	0.0060
GO:0,050,896	Response to stimulus	5812	14	2.2	0.0095
GO:0,023,052	Signaling	4569	12	2.3	0.0095
GO:0,002,376	Immune system process	573	4	6.3	0.0109
GO:0,051,716	Cellular response to stimulus	5003	12	2.1	0.0159
GO:0,006,952	Defense response	257	2	6.9	0.0489

^*^After false discovery rate correction following Benjamini and Hochberg

## Discussion

The genome of the pygmy right whale (*Caperea marginata*) allowed us to make a first genetic analysis of a species which is so far nearly unknown to science. Due to its small body size and unique monotypic placement within the mysticetes, the pygmy right whale represents a promising target species to better understand the general evolution of rorquals and to analyze cancer resistance in baleen whales.

### Genome features, diversity, and demography

Despite our best efforts to improve the assembly, we did not reach chromosome-level continuity. This could have been caused by either high levels of DNA fragmentation or by a high repeat content, which is typical for baleen whales [73]. Furthermore, there may be unique features in the repetitive sequences of the pygmy right whale genome that further hindered a more continuous assembly. Chromatin-based assembly methods (Hi-C) [74] will likely increase the assembly continuity but rely on fresh tissue samples that are very difficult to get given the elusive nature of the pygmy right whale. Completeness scores also showed that some core genes were fragmented or missing. However, assembly continuity mostly affects the analysis of structural genome changes or gene copy numbers and is therefore unlikely to affect our downstream analyses.

The newly constructed genome allowed us to assess the genetic diversity and to model the previously unknown demographic history. The level of genome-wide heterozygosity is comparable to that of the blue and North Atlantic right whales [75], but higher compared to other mysticetes [20, 75], although comparing genetic diversity alone does not allow conclusions about the well-being of a species [75, 76]. Our demographic model showed a population trajectory over time that is similar to other baleen whales, starting from a high abundance around the Late-Pleistocene Transition (2.6 Mya) and slowly declining in Ne over time until reaching a lower, but stable population size after the Mid-Pleistocene Transition (1.6 Mya–700 kya) [20, 77]. After this point in time, the trajectory shows no indications of an influence from major climatic oscillations that would have consequently affected marine circulation and productivity [78].

### Revision of the rorqual phylogeny

The multispecies coalescent (MSC) phylogeny presented in this study is based on fragments of a whole-genome alignment (WGA) that includes data from twelve different whale species including nearly all extant members of the *Mysticeti.* This analysis resulted in an overall well-supported topology that unequivocally placed the pygmy right whale as expected from previous studies [13, 14, 18, 19] but showed a high degree of phylogenetic conflicts for the gray whale (*Eschrichtius robustus*). Other sources of homologous data like, e.g., single-copy orthologous sequences (SCOS) or other methods of gene tree conflation resulted in different placements of the gray whale and even more phylogenetic conflicts across all tree nodes.

These conflicts, depicted by the distribution quartet scores, were found to be nearly even at the branch positioning the gray whale. Because the phylogenomic tree based on WGA fragments had the fewest amount of conflicts in the entire tree, we consider the pairing of the gray whale together with the fin and humpback whale as the most parsimonious explanation of baleen whale evolution, supporting previous findings by [19, 20], and [73]. Nevertheless, we still would not consider this placement as definitive, nor the topology being resolved as a bifurcating event given the nearly equal frequency of conflicting signals. Instead, we suggest that the relationship of the gray whale is best depicted as a polytomy. In the past, such polytomies were thought to be a consequence of a lack of molecular data or taxon sampling and were treated as soft polytomies with the expectation that an increase of data would eventually lead to highly resolved bifurcating trees [79]. However, in this case, only a small increase in the data remains possible and thus we think it is unlikely that the resolution improves in the future. Thus, we consider this polytomy as a hard polytomy that reflects the actual biological history of a rapid radiation of rorquals at the beginning of their divergence 15–25 million years ago (Fig. 3).

Two scenarios can cause sub-trees to deviate from the overall species tree: incomplete lineage sorting (ILS) and introgression. While ILS occurs randomly at predictable frequencies that will not overshadow the true species tree topology [80], introgression might, depending on its extent, obscure the true topology. Within baleen whales, ongoing introgression seems unlikely, given the lack of runs of shared phylogenetic signals across the genome (Fig. 1C). The nearly equal occurring frequencies of alternative topologies in the quartet score analysis (quartet 4, Fig. 1B) would, according to the neutral MSC model, also indicate dominant ILS within the conflicting signals [80]. However, our QuIBL results point out a high amount of ancient introgression (Additional file 1: Table S2) which could have been overshadowed by ILS and recombination over time. These ancient introgression events could also, together with the number of discordant trees caused by ILS, result in a false topology of bifurcating branches that cannot be resolved unless the entire history of introgression events could be unscrambled. Such an attempt to remove putative signals of introgression in a similar case of mbuna cichlids did not alter the distribution of conflicts for the problematic branches [81]. Therefore, choosing between one of the three possible topologies might not be possible, which supports our finding that the evolution of rorquals is best described as a hard polytomy.

Finally, a hard polytomy should not be confused with an unresolved phylogeny, as it is only unresolved in the sense of a strictly bifurcating tree. Yet, with the discover of many hard polytomies like presented here [81–83] we would like to stress that evolution must not be a bifurcating process by all means and that cases like the presented radiation in rorquals are best depicted as an evolutionary network [84].

### Identification of cancer-related genes

Our pairwise selection analysis between large and small-bodied mysticetes like the pygmy right whale resulted in a set of 210 candidate genes that might be related to body size and hence cancer resistance following the idea behind Peto’s paradox [21]. Within this set, we found six genes with similar signals of positive selection across all three pairs of whales. All except of one were already known to cancer research due to correlations between their over- or under-expression and cancer development (Table 2 [66–70]). The proline-rich protein LOC124907494 (human) is to our knowledge unknown to cancer research and shows the most non-synonymous mutations in large baleen whales, therefore, representing an interesting target for further research. Within our six selected genes, two were already described in greater detail, namely CLEC2B and REP15 [68, 69]. CLEC2B is a member of the C-type lectin domain family 2 and was formally described as activation-induced C-type lectin (AICL) [71]. It is encoded by the natural killer (NK) gene complex proximal CD69 and its transcription is increased during lymphocyte activation [71]. Recently, many studies have highlighted its association with various types of cancer [68, 85] and it is assumed to be connected to the immune response to cancer through ferroptosis activation [68]. Its positive selection in large-bodied baleen whales might represent adaptations to this complex to better control the activation and migration of lymphocytes when encountering cancer. REP15 is the effector of the Rab15 GTPases which are assumed to control vesicular traffic in neuronal tissue [69], a function possibly involved in the adaptions to increase body size. However, a multi-omics analysis also suggested REP15 as a colorectal cancer-specific driving gene [86], and it was shown to interact with other Rab proteins [69] of which many are discussed to be involved in tumorigenesis because of its potential role in signal transduction to stimulate progression and invasion into other areas [87].

Similar implications of adaptions in cell signaling were also found in a gene-enrichment analysis performed on the complete set of 210 candidate genes. Apart of enriched functions involved in signal transduction, we also detected other general GO terms to be enriched like, e.g., cell communication, immune system processes, and defense responses (Table 3). The most enriched function in our analysis was the G-protein-coupled receptor (GPCR) signaling pathway. GPCRs are the largest class of surface-bound receptors with around 900 representatives involved into a variety of basic physiological functions and a growing body of literature describes their diverse implications in cancer initiation, development, survival, and migration [88]. Hence, there is a high potential that adaptions in this pathway resulted in an increased cancer resistance in whales.

Our comparisons between pairs of mysticetes with diverging body sizes revealed that only few genes featured similar signals of positive selection. Furthermore, the here presented enriched functions represent rather general terms that do not allow further specification of exact pathways. One explanation for this might be that large body sizes emerged several times during the evolution of baleen whales, with similar selective pressures towards size increase, but different specific adaptions fixed within the genotypes. Paleontologists have often reported a discrepancy between the size of baleen whale fossil records and extant species [89]. Until 10–12 Mya, mysticetes are considered to have remained less than 10 m long [90], being more comparable to the here featured pygmy right whale than to other gigantic representatives. By combining size records with a phylogenetic framework, Slater et al. (2017) [90] simulated the evolution of large body sizes in baleen whales and located the emergence of gigantism within 5–3 Mya, a period defined by the onset of Northern Hemisphere glaciation and the loss of diversity in small-bodied mysticetes [19]. Increasing positive selection towards larger body sizes may have therefore affected all lineages of baleen whales, resulting in a convergently evolved gigantism within the right whales and multiple clades of rorquals. This evolutionary history would explain the lack of shared candidate genes highlighted in pairwise selection comparisons thus far, including the here presented analysis (Table 2) [22, 24, 25]. Nevertheless, while adaptions in cancer resistance may not be specifically conserved in large-bodied baleen whales, it is still noteworthy that adaptions happened in the same functional categories, because genes belonging to general GO terms like signal transduction, cell communication, immune system processes and cell defense mechanisms were highlighted in every related study thus far [22, 24, 25]. Therefore, focusing efforts to these specific functions might help understanding the whale specific cancer resistance in the future.

## Conclusions

In this study, we presented the first de novo genome of the pygmy right whale, the smallest baleen whale species, and the only member of an otherwise extinct family of whales. The genomic data from this species was used to update the baleen whale phylogeny, revealing a hard polytomy between the gray whale and other related rorquals caused by high amounts of introgression at the beginning of their radiation. Additionally, the new genome was included in a genome-wide comparison of selection rates to identify genes related to large body size and hence cancer resistance in mysticetes, resulting in only a small set of common candidate genes supporting a more convergent evolution of gigantism in baleen whales.

## Methods

### Sampling, DNA isolation, and sequencing

Tissue samples were collected by Prof. Dr. Eric Harley from an individual that was washed ashore at the coast of Simonstown, South Africa, in 1993. Samples were subsequently stored in 70% ethanol at − 20 °C. DNA was extracted from approximately 100 mg of tissue using a standard phenol–chloroform-isoamylalcohol protocol [91]. A 10X Genomics Chromium library was constructed by SciLifeLab and a subsequent sequencing yielded approximately 368.6 million paired/linked 150 bp long Illumina reads (~ 23-fold coverage). To generate long reads, four SMRTbell libraries were constructed following the instructions of the SMRTbell Express Prep kit v2.0 (Pacific Biosciences, Menlo Park, CA). Four SMRT cell sequencing runs were performed in “Continuous Long-Read” (CLR) mode on the Sequel System II with the Sequel II Sequencing Kit 2.0 resulting in ~ 1.9 million reads of 20 kbp or more (~ 35-fold coverage).

### Genome assembly and annotation

A whole-genome assembly was performed by Supernova v2.1.1 [92] using the linked short-read data. The intermediate assembly was scaffolded with Sspace-LongRead v1-1 [93] using the long-read data. Gap-closing was performed with TGS-GapCloser v.1.2.0 [94] utilizing the long-read data as well. Polishing was done by first mapping the linked short reads onto the gap-closed assembly with Bowtie 2 v.2.4.5 [95]. The mapping file was filtered for duplicates with the Picard v2.21.2–0 toolkit (https://broadinstitute.github.io/picard/) before being used in variant calling using DeepVariant v.1.3.0 [96]. Resulting variants were utilized to generate a polished assembly by calling consensus sequences with Bcftools v.1.12 [97]. Eventually, gene set completeness was assessed with Busco v5.3.2 [98] by testing the OrthoDB gene sets of *Cetartiodactyla*, *Laurasiatheria*, and *Mammalia* [99].

To test whether the long-read data would result in more continuously assembled contigs when used for the initial assembly, we tested them with different specialized software, namely: Canu v2 [100], wtdbg2 [101], and Flye v2.3.3 [102] following respective user recommendations. We conducted the same downstream efforts to scaffold and polish the resulting assemblies as described above for the final assembly. However, since none of the long-read assemblies reached similar continuity compared to the linked short-read assembly, we eventually decided to use the latter in all following analyses.

Repeats were identified using Repeatmodeler v2 (www.repeatmasker.org) and found repeats were merged with the *Cetartiodactyla* repeat database from RepBase [103]. Resulting dataset was used by Repeatmasker v4.1 (www.repeatmasker.org) to mask repeats within the de novo assembly. A first annotation of the genome was performed with the GeMoMa pipeline [104] which identifies genes based on homologous information provided by different annotations of related individuals. Doing so, we collected annotations for other *Cetacea* on various different databases. A complete list of all used annotations can be found in Additional file 1: Table S6 [22, 26, 28–31, 75, 105]. Eventually, found annotations were functionally annotated using InterProScan v5 [32].

### Genome diversity and demography

Genome-wide diversity was estimated by the means of genome-wide heterozygosity (HE). Short reads produced by the 10X Chromium platform were therefore trimmed for adapter sequences using the Longranger v.2.2.2 toolkit (https://support.10xgenomics.com) before they were mapped onto the masked de novo assembly with BWA-mem v0.7.17-r1188 (http://bio-bwa.sourceforge.net). Variances were called by BCFtools v1.12 mpileup [97] with the respective “-c” flag and minimal mapping- and base-quality cutoffs of 30. These genotypes were additionally filtered for a too divergent read coverage (> threefold and < 0.3-fold of the expected mean coverage) and for sites with a too high proportion of missing data (5%) using BCFtools v1.12 filter [97]. Eventually, genome-wide heterozygosity was inferred as the proportion of heterozygous genotypes compared to the total genotype set including monomorphic sites.

A first model of the demographic past of the species was constructed with the pairwise sequentially Markovian coalescent (PSMC) framework [33] using the repeat masked genome sequences generated above, a standard of 64 atomic intervals (− *p* = 4 + 25*2 + 4 + 6) and a mutation rate of 1.39 × 10^−8^ per site per generation [24]. Because no generation time estimates exist for the pygmy right whale, we used the generation time of the minke whale as an approximation (22.1 years) [34]. To assess potential variances, 100 bootstrap iterations were performed and were depicted as thinner lines.

### Phylogenomics

Phylogenomic reconstruction was conducted based on a whole-genome alignment approach. Therefore, we collected eleven assemblies of other baleen whale species from either the NCBI genome (https://www.ncbi.nlm.nih.gov/genome/) or from DNA Zoo (https://www.dnazoo.org/assemblies). A graphical depiction of our entire phylogenetic workflow is presented in the Additional file 1: Fig. S6. A complete list of all utilized data can be found in Additional file 1: Table S6 [20, 22, 24, 28–31, 75, 105–107].

To generate whole-genome alignments, we followed the overall workflow presented in [108] and most of the respective tools are available on github.com (hillerlab/GenomeAlignmentTools). Briefly, pairwise alignments between the repeat-masked reference genome of the common bottlenose dolphin (*Tursiops truncatus*) (NCBI genome: GCA_011762595.1) and individual baleen whale genomes were constructed using lastz v1.04.15 [35] with the default scoring matrix and the following parameter: “*K* = *2400, L* = *3000, Y* = *9400, H* = *2000*”. Co-linear alignment chains were constructed with axtChain [36]. RepeatFiller[37] was used to further align repetitive regions and ChainCleaner [38] with parameters “*LRfoldThreshold* = *2.5 -doPairs -LRfoldThresholdPairs* = *10 -maxPairDistance* = *10,000 -maxSuspectScore* = *100,000 -minBrokenChainScore* = *75,000*” was used to improve alignment specificity. Alignment chains were converted to alignment nets with ChainNet and nets were filtered with NetFilterNonNested.perl, where we applied an overall score threshold of 100,000 and kept syntenic or inverted nets with scores ≥ 5000. Filtered alignment nets were used to compute a whole-genome alignment (WGA) with Multiz-Tba [109] and all unaligned regions were removed from the final alignment.

To generate WGA fragments, we first extracted single species fasta files from the alignment and removed all gaps and ambiguous sites with Bedtools v.2.30 [110]. Bedtools was used to create a dictionary of positions where a gap or ambiguous site occurred in one of the individuals to remove all respective positions in all individuals subsequently. We then generated 20-kbp-sized WGA fragments using the scripts presented in [111]. To evaluate ideal fragment sizes, we conducted an approximately unbiased test (Additional file 1: Fig. S1) [112] as described in Árnason et al. (2018) [20] by testing different placements of the pygmy right whale within the topology of in Árnason et al. (2018) [20]. We further filtered for too conserved and too variable fragments by removing the 5% most variable and least variable fragments, given the maximum likelihood distance inferred using IQTree v.2.1.2 [113]. For each fragment, a phylogenetic tree was constructed using IQTree with 1000 bootstrap replications before summarizing them to a consensus species tree with Astral-III v.5.7.3 [114]. In doing so, we annotated branches with quartet scores and posterior probabilities.

Branch lengths were calculated from a maximum likelihood analysis based on single copy orthologous sequences (SCOS) and a respective pipeline regarding the generation of SCOS datasets and downstream phylogenetic analyses can be found on github.com (mag-wolf/GEMOMA-to-Phylogeny). Because the resulting set of SCOS is used in multiple downstream analyses (tree calibration and SCOS consensus tree) we included multiple species of *Odontoceti* as well as the hippopotamus (GCA_023065835.1), camel (GCA_000803125.3), and cow (GCA_002263795.3) as outgroups to fulfill the requirements of all analyses. We collected publicly available genome assemblies and protein data as listed in Additional file 1: Table S6. We re-annotated all collected assemblies using the GeMoMa pipeline [104] by conducting homology-based annotations using all available proteomes. SCOS were called by OrthoFinder v.2.5.2 [115] using default parameters and the “MSA” method for gene tree inferences. Gene alignments were constructed using Mafft v.7.475 [116]. To avoid using misaligned or uninformative alignments we applied cutoffs of not more than 40% variable sites and more than 5% variable sites. Alignments were concatenated to a single matrix using FASconCAT-G v1.04 [117]. The concatenated matrix was trimmed with ClipKit v.1.1.3 [118] for informative and conserved sites, allowing an additional gap trimming with the “-m kpic-smart-gap” flag. Eventually, the resulting matrix was used to calculate branch lengths with IQTree.

Because quartet scores regarding the placement of the gray whale were exceptionally even, we evaluated if they were caused by reginal conflicting sites or if conflicts were evenly distributed across the genome. To do so, all WGA fragments that originated from the reference chromosome_1 were further divided into 1 kbp windows. We assessed their number of informative sites and excluded them when containing less than 50 informative sites. Quartet scores per 20 kbp WGA fragment were then calculated based on the 1kbp windows using IQTree and Astral-III as described above.

To decipher, whether discordant WGA fragment trees originate from incomplete lineage sorting (ILS) or introgression, we used QuIBL (“quantified introgression via branch lengths”) as described in Edelman et al. (2019) [39]. We used 1000 randomly selected trees from the set of filtered 20kbp WGA fragment trees and applied a likelihood threshold of 0.01, 50 EM steps, and a shrinking factor of 0.5. The resulting output can be found in Additional file 1: Table S2. Doing this analysis, QuIBL applies a Bayesian information criterion test (BIC) for both possible scenarios of “ILS” and “ILS + introgression”. To decide if a signal truly originates from introgression, we used a strict cutoff of ΔBIC <  − 10.

Consensus networks were generated using SplitsTree 4 [119] and all filtered 20-kbp WGA fragment trees by evaluating different cutoffs of conflicting edges. In doing so, we tested cutoffs between 7 and 30% and eventually used 12% for the final depiction of conflicts.

We further tested the performance of alternative sources of homologous information and alternative construction methods. First, we constructed a consensus tree based on all SCOS trees individually using the workflow described above and evaluated their quartet score distribution across the tree using IQTree and Astral-III. Second, we conducted a multispecies coalescent (MSC) tree inference based on single nucleotide polymorphisms (SNPs). These SNPs were called from available short-read data of nine baleen whale species (Additional file 1: Table S6) mapped onto the bowhead whale (*Balaena mysticetus*) reference genome [22] using the same workflow to generate vcf files as described for the pygmy right whale alone during the genetic diversity assessment. The filtered vcf file that still contained monomorphic sites was used to generate biallelic SNPs with Vcftools v.0.1.16 [120]. SNPs were pruned for linkage disequilibrium using the Bcftools plugin “ + *prune*” applying a *r*2 = 0.9 cutoff. Coalescence inference was done with SVDquartets [121] which is implemented in PAUP 4.0a (Windows build 169). In doing so, we used the QFM algorithm [122] and conducted 1000 bootstrap replications. A sliding window approach was used to generate a subset of trees with IQTree using 50 SNPs per window. The set of trees was eventually used for quartet score evaluation of the MSC tree with Astral-III.

A dated tree was constructed by calibrating the topology of the consensus WGA tree presented in the main figure. To include more calibration points, we extended the topology with species of *Odontoceti* as well as hippopotamus, camel, and cow by including the orthologs of all respective species in a SCOS consensus tree as described above. Resulting topology was calibrated with MCMCtree which is part of the PAML 4.9 package [123] using five calibration points (Additional file 1: Table S3) and the concatenated SCOS matrix.

### Selection analyses

Genes putatively involved in cancer resistance in baleen whales were identified by conducting pairwise comparisons of non-synonymous and synonymous substitution rates (Ka/Ks) between a large and a small-bodied baleen whale. To find phylogenetic independent pairs that at the same time maximize size differences within pairs, phylogenetic targeting was applied [72] by specifying the bottlenose dolphin as outgroup and using size and body mass data listed in Additional file 1: Table S4 [45–65]. Candidate pairs were identified based on the tree topology depicted in Fig. 1 and on the standardized summed score.

To collect as many informative orthologs between the six whales as possible, we constructed a second set of SCOS including only the candidate whales as well as the human reference genome GRCh38. We re-run the GEMOMA-to-Phylogeny pipeline, as described above in the phylogenetic section. In doing so, we inferred SCOS between all candidate whales together with the human genome GRCh38. To ensure that alignments were constructed without frameshifts, we first translated nucleotide sequences to amino acid sequences using the EMOSS v6.6.0.0 transeq [124] tool before generating multiple sequence alignments with Mafft. Amino acid alignments were then converted back to codon alignments using Pal2Nal v14 [125] using the “-nogap” function to remove gaps as well as in-frame stop codons. To avoid alignment errors being accounted for in downstream Ka/Ks analyses, we removed alignments with the five percent topmost genetic distances using the maximum likelihood distance calculated by IQTree. Filtered codon alignments were then converted into axt files using AXTConverter [126] before inferring pairwise non-synonymous and synonymous substitution rates with KaKs_Calculator v2 [126]. We screened our results for signals of putative positive selection (Ka/Ks > 1, for the distribution of Ka/Ks see Additional file 1: Fig. S7) and inferred a total as well as a shared set of candidate genes which we further functionally annotated via BLASTn against the general “db” database using a cutoff of 1e^−25^.

A gene enrichment analysis was conducted by extracting the human orthologs of all putatively positive selected candidate genes and comparing their functions against the annotation of the human genome reference GRCh38 provided by NCBI (https://www.ncbi.nlm.nih.gov/ projects/genome/guide/human/). Enrichment of functions was inferred using the “parentchild” algorithm implemented in the R-package TopGO [127]. Significance values were corrected for false discovery rates after Benjamini and Hochberg using the R package p.adjust. We removed GO terms with only a single occurrence to avoid taking artifacts into account. GO terms exceeding a corrected *p*-value of > 0.05 were eventually used to construct a “TreeMap” with REVIGO [128].

## Supplementary Information

**Additional file 1: Fig. S1.** An approximately unbiased (AU) test for increasing whole-genome alignment fragment sizes. **Fig. S2.** Consensus networks of baleen whales based on whole-genome alignment fragments and different thresholds. **Fig. S3.** Phylogenomic analysis of baleen whales using protein coding sequences of shared single copy orthologous sequences. **Fig. S4.** Phylogenomic analysis of baleen whales using single nucleotide polymorphisms. **Fig. S5.** Consensus network of Cetacea evolution based on single copy orthologous sequences. **Fig. S6.** Pipeline depicting the process of generating all phylogenomic trees. **Fig. S7.** Distribution of Ka/Ks values over all tested orthologs. **Table S1.** Repeat content of the pygmy right whale assembly. **Table S2.** QuIBL results for all triplets resulting from combining rorquals species affecting the placement of the gray whale. **Table S3.** Calibration points used in the date phylogeny. **Table S4.** Body mass data used for phylogenetic targeting. **Table S5.** Maximal pairs inferred from the phylogenetic targeting analysis. **Table S6**. Used data featured in this study including assemblies, short read archives and proteomes from other *Cetacea or Cetartiodactyla*.

## Data Availability

Raw sequencing reads have been deposited at the National Center for Biotechnology Information under the BioProject PRJNA856827. The assembled genome sequence of the pygmy right whale is deposited as Genome: JANTQK000000000, BioSample: SAMN29592900. All secondary data, including sequences, alignments, and tree files are uploaded to a Dryad repository: https://doi.org/10.5061/dryad.9zw3r22j0. The pipeline used to create SCOS datasets and phylogenetic trees is available at: https://doi.org/10.5281/zenodo.7740016 and https://github.com/mag-wolf/GEMOMA-to-Phylogeny. All data generated or analyzed during this study are included in this published article, its supplementary information file, and publicly available repositories. Additional data related to this paper may be requested from the authors.

## Abbreviations

BIC: Bayesian information criterion
BiK-F: Biodiversity and Climate Research Centre
bp: Base-pairs
BUSCO: Benchmarking Universal Single-Copy Orthologs
CLEC2B: C-type lectin domain family 2 member B
Gbp: Giga-base-pairs
GO: Gene Ontology
GPCR: G-protein-coupled receptor
HE: Heterozygosity
HGNC: HUGO Gene Nomenclature Committee
HUGO: Human Genome Organization
ILS: Incomplete Lineage Sorting
IUCN: International Union for Conservation of Nature
kbp: Kilo-base-pairs
kya: Thousand years ago
Mbp: Mega-base-pairs
ML: Maximum likelihood
MSC: Multi-species coalescent
Mya: Million years ago
NCBI: National Center for Biotechnology Information
PSMC: Pairwise sequentially Markovian coalescent
QuIBL: Quantified introgression events via branch lengths
REP15: RAB15 effector protein
SCOS: Single copy ortholog sequences
SNP: Single nucleotide polymorphism
TBG: Translational Biodiversity Genomics
TSG: Tumor suppressor gene
WGA: Whole-genome alignment
