# Supplementary material for: The genome of the pygmy right whale illuminates the evolution of rorquals
Source: BMC Biol. 2023 Apr 12;21:79. doi: 10.1186/s12915-023-01579-1 (PMC10091562; doi:10.1186/s12915-023-01579-1)
Supplement: Supplementary file 1 — Additional file 1: Fig. S1. An approximately unbiased (AU) test for increasing whole-genome alignment fragment sizes. Fig. S2. Consensus networks of baleen whales based on whole-genome alignment fragments and different thresholds. Fig. S3. Phylogenomic analysis of baleen whales using protein coding sequences of shared single copy orthologous sequences. Fig. S4. Phylogenomic analysis of baleen whales using single nucleotide polymorphisms. Fig. S5. Consensus network of Cetacea evolution based on single copy orthologous sequences. Fig. S6. Pipeline depicting the process of generating all phylogenomic trees. Fig. S7. Distribution of Ka/Ks values over all tested orthologs. Table S1. Repeat content of the pygmy right whale assembly. Table S2. QuIBL results for all triplets resulting from combining rorquals species affecting the placement of the gray whale. Table S3. Calibration points used in the date phylogeny. Table S4. Body mass data used for phylogenetic targeting. Table S5. Maximal pairs inferred from the phylogenetic targeting analysis. Table S6. Used data featured in this study including assemblies, short read archives and proteomes from other Cetacea or Cetartiodactyla. [file 12915_2023_1579_MOESM1_ESM.pdf]

Supplementary Materials for

The genome of the pygmy right whale illuminates the evolution of rorquals.

Magnus Wolf[1,2], Konstantin Zapf[1,2], Deepak Kumar Gupta[3], Michael Hiller[1,3], Úlfur Årnason[4,5], Axel Janke[1,2,3]

*Corresponding author: Magnus Wolf; Email: Magnus.Wolf@senckenberg.de

This PDF file includes:

Figs. S1 to S7

- **Fig. S1 An approximately unbiased (AU) test for increasing whole-genome alignment fragment sizes.**
- **Fig. S2 Consensus networks of baleen whales based on whole-genome alignment fragments and different thresholds.**
- **Fig. S3 Phylogenomic analysis of baleen whales using protein coding sequences of shared single copy orthologous sequences**.
- **Fig. S4 Phylogenomic analysis of baleen whales using single nucleotide polymorphisms**.
- **Fig. S5 Consensus network of Cetacea evolution based on single copy orthologous sequences**.
- **Fig. S6 Pipeline depicting the process of generating all phylogenomic trees.**
- **Fig. S7 Distribution of Ka/Ks values over all tested orthologs.**

Tables S1 to S6

- **Table S1 Repeat content of the pygmy right whale assembly.**
- **Table S2 QuIBL results for all triplets resulting from combining rorquals species affecting the placement of the gray whale.**
- **Table S3 Calibration points used in the date phylogeny.**
- **Table S4 Body mass data used for phylogenetic targeting.**
- **Table S5 Maximal pairs inferred from the phylogenetic targeting analysis.**
- **Table S6 Used data featured in this study including assemblies, short read archives and proteomes from other *Cetacea* or *Cetartiodactyla*.**

Supplementary Figures

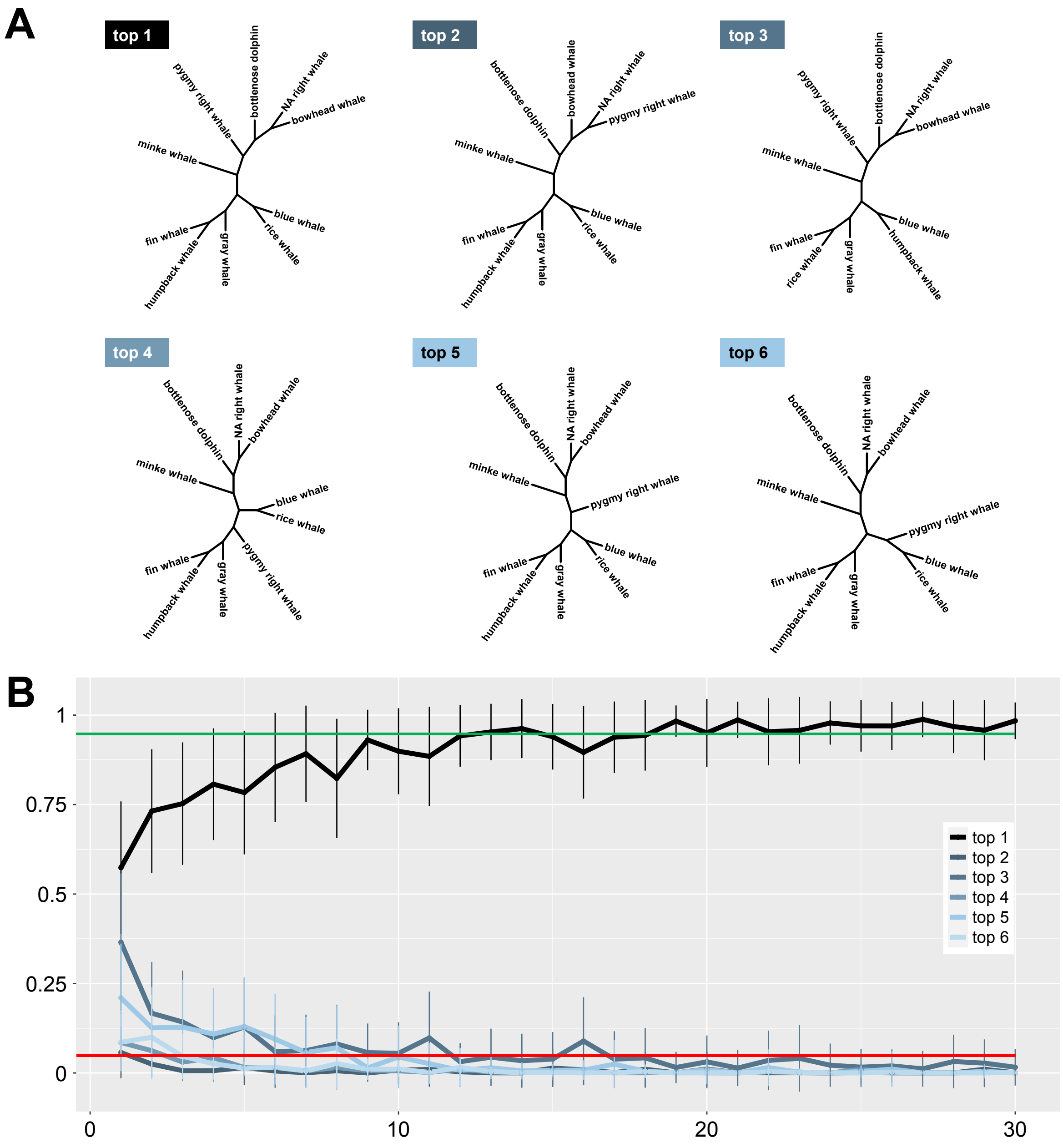

Fig. S1 An approximately unbiased (AU) test for increasing whole-genome alignment fragment sizes. A Different topologies (top1-top6) evaluated by testing different placements of the pygmy right whale (*Caperea marginata*). **B** Distribution of AU values for increasing fragment sizes given different topologies. The green and red line mark pAU=0.05 intervals at which alternative hypothesis could be rejected. Based on this AU test, a fragment size of 20 kbp was chosen for downstream analyses.

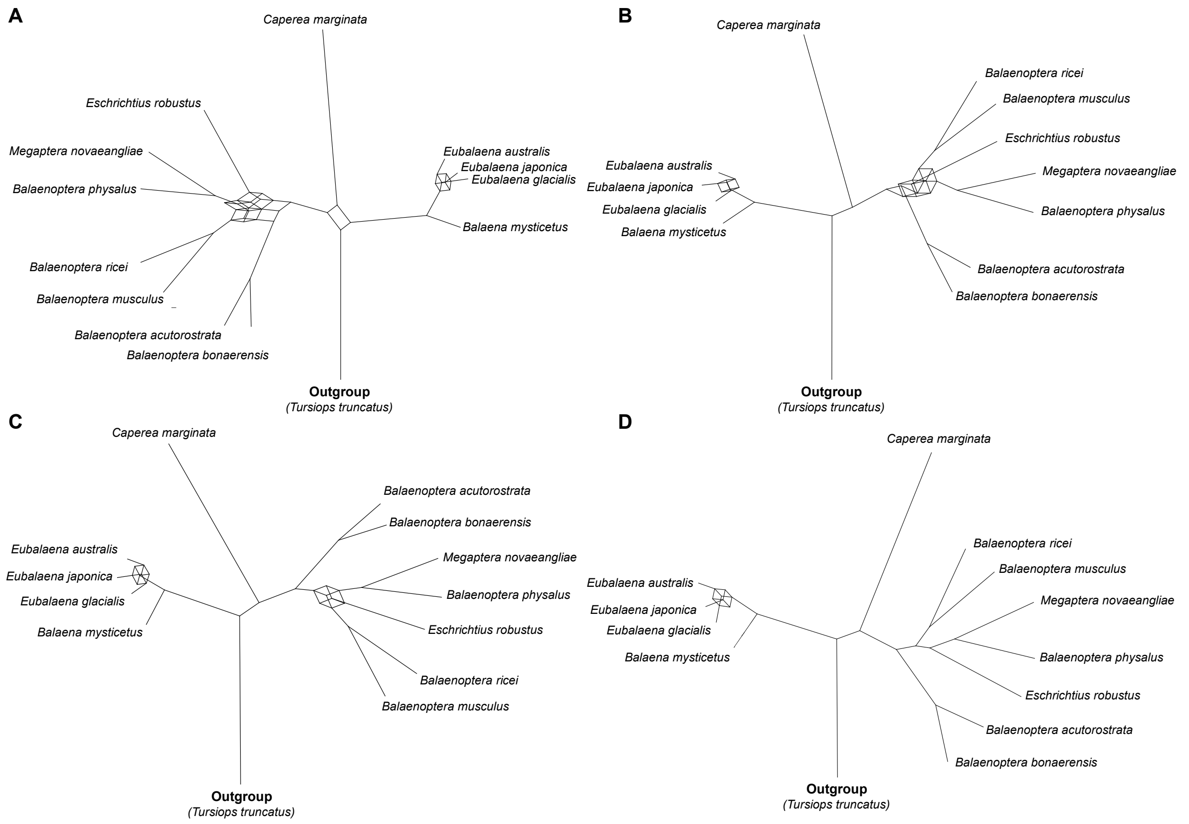

Fig. S2 Consensus networks of baleen whales based on whole-genome alignment fragments and different thresholds. Thresholds were lowered stepwise starting from 30% until reaching 5%. **A**. 5% - 7%, **B**. 7% - 11%, **C**. 11% - 12%, **D**. 12% - 30%.

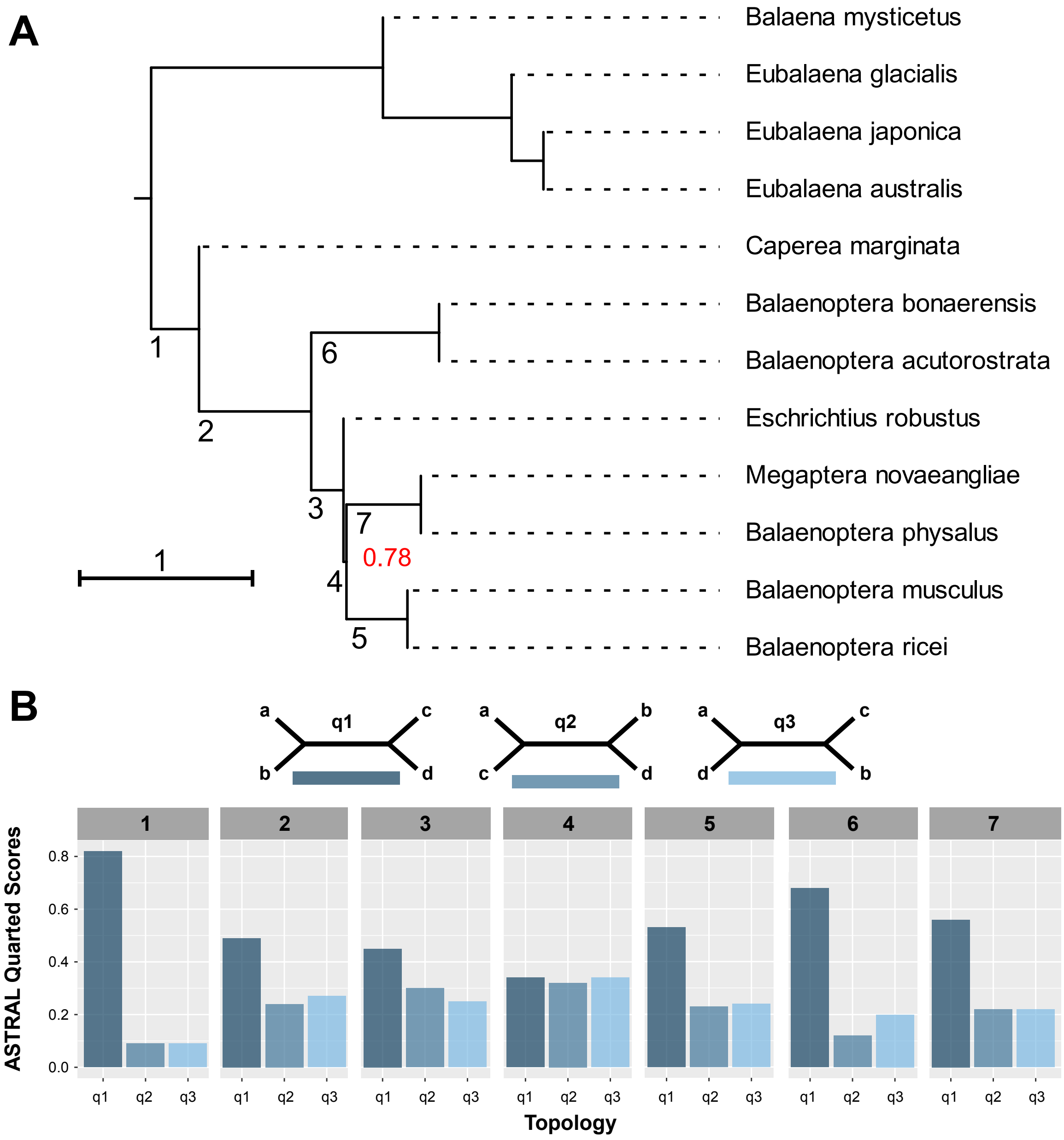

Fig. S3 Phylogenomic analysis of baleen whales using protein coding sequences of shared single copy orthologous sequences. **A**. Phylogenomic multi-species coalescent (MSC) tree conflated from 563 trees that were each constructed from SCOS. All branches except the branch separating (fin whale and humpback whale) and (blue whale and rice whale) received maximum bootstrap support while the latter received 78% support. The pygmy right whale was placed at the base of the rorquals, and the gray whale was placed with a short branch at the base of other large rorquals. **B**. Quartet scores of different branches across the MSC tree. Branches 1 - 7 were analyzed for the amount of gene trees supporting one of the three possible unrooted topologies (q1 - q3). All branches featured substantial conflicting signals while branch 4 received nearly equal frequencies for each alternative topology similar to what was shown in Fig 1 of the main manuscript.

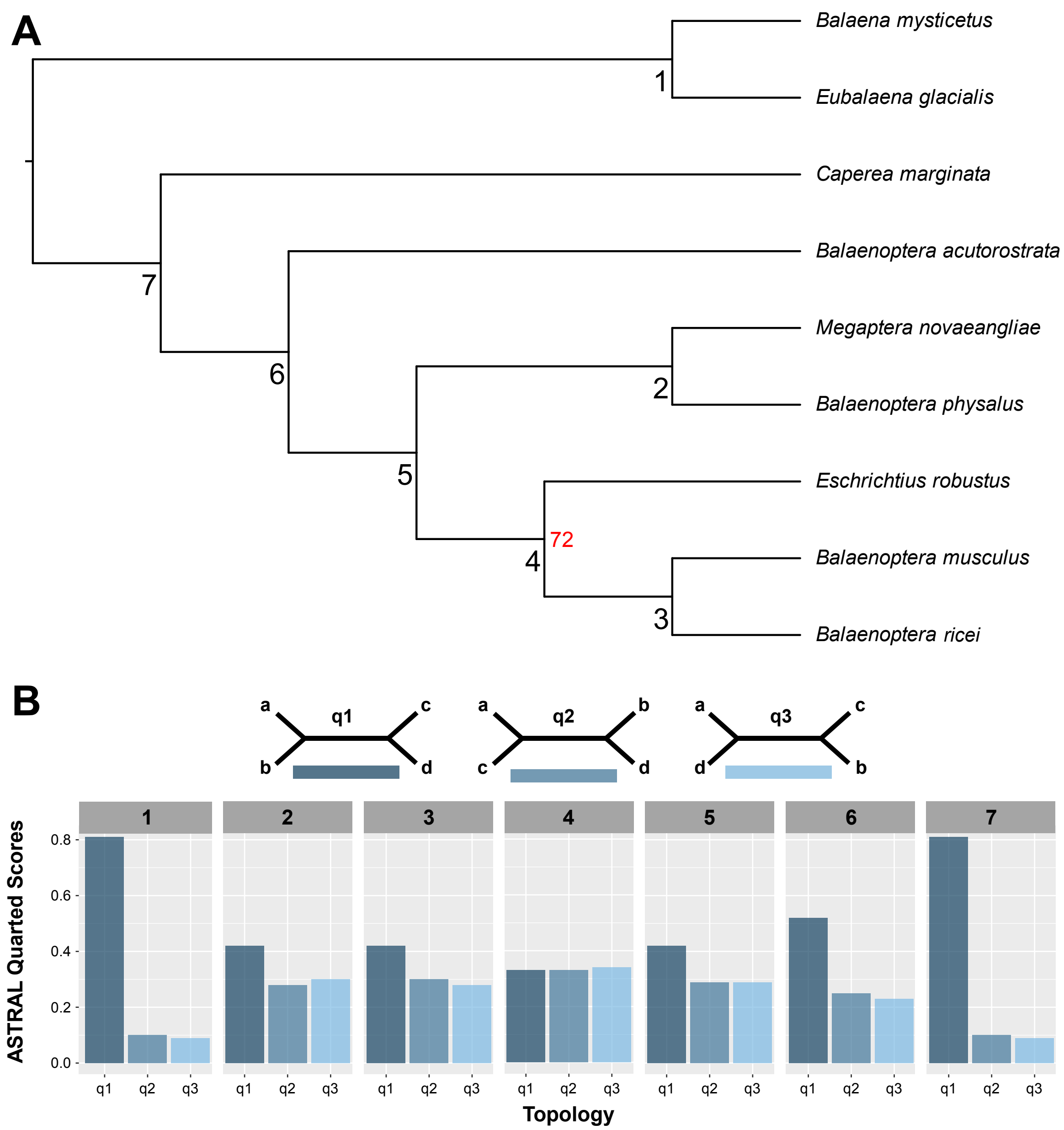

Fig. S4 Phylogenomic analysis of baleen whales using single nucleotide polymorphisms. **A**. Phylogenomic multi-species coalescent (MSC) tree directly inferred from 1.7 million SNPs. All branches except the branch grouping the gray whale with the blue and rice whale received 100% bootstrap support while the latter received 72%. The pygmy right whale was placed at the base of the rorquals, and the gray whale was grouped together with the blue and rice whale forming sister clade to the fin and humpback whale. **B**. Quartet scores of different branches across the MSC tree were inferred using trees constructed from 50 SNP windows. Branches 1 - 7 were analyzed for the amount of trees supporting one of the three possible unrooted topologies (q1 - q3). All branches featured substantial conflicting signals while branch 4 received nearly equal frequencies for each alternative topology similar to what was shown in Fig 1 of the main manuscript.

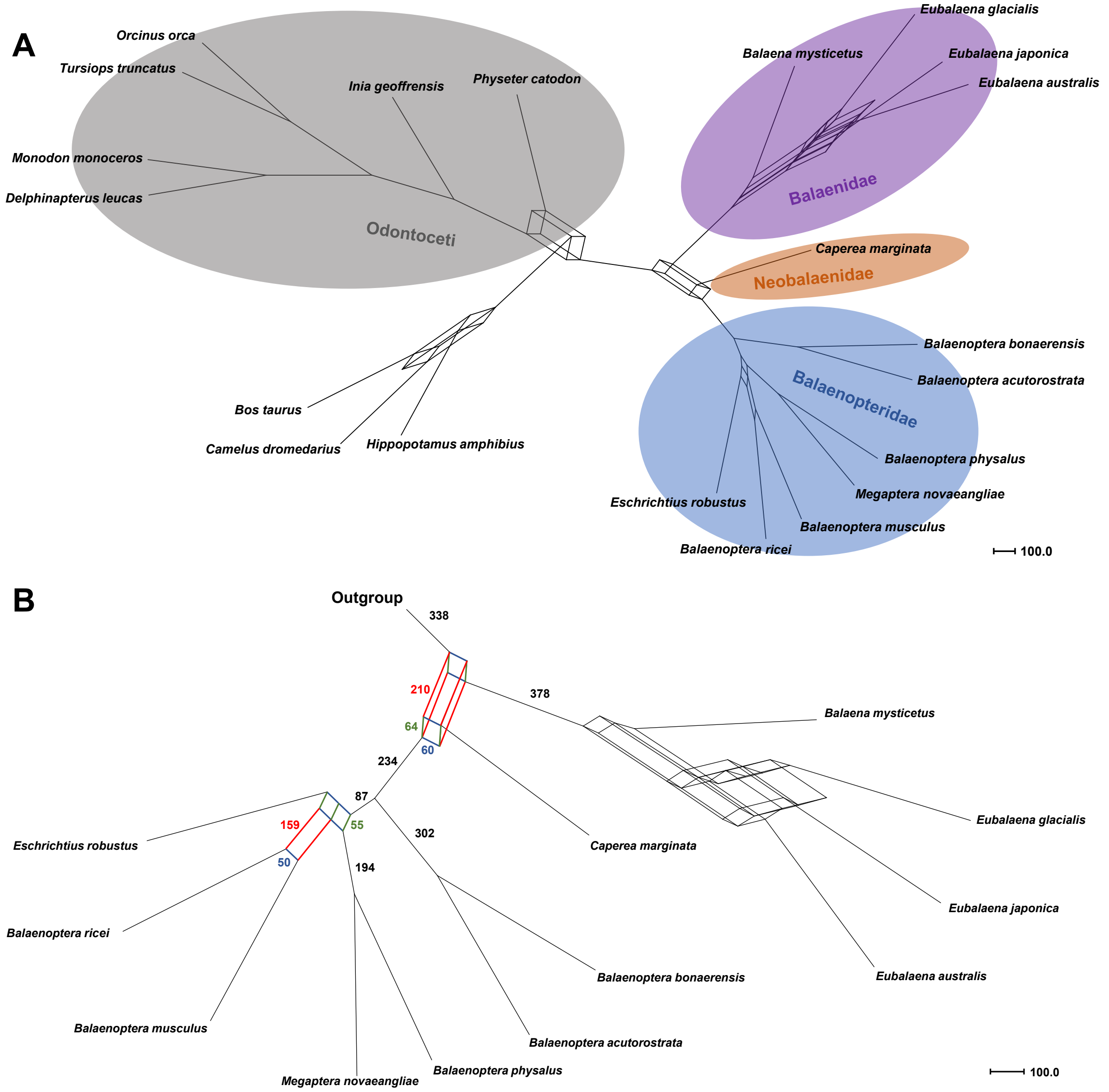

Fig. S5 Consensus network of Cetacea evolution based on single copy orthologous sequences. The network was conflated from gene trees that were constructed from 563 SCOS and a 12% threshold was used to depict conflicts. Extensive phylogenetic conflicts characterize the placement of the gray whale consistent with branch 4 of the main phylogenomic analysis. Additional conflicts were found at the placement of the pygmy right whale, at the base of the right whale divergence and at the branch separating toothed whales (*Odontoceti*) and baleen whales (*Mysticeti*), although they were less even.

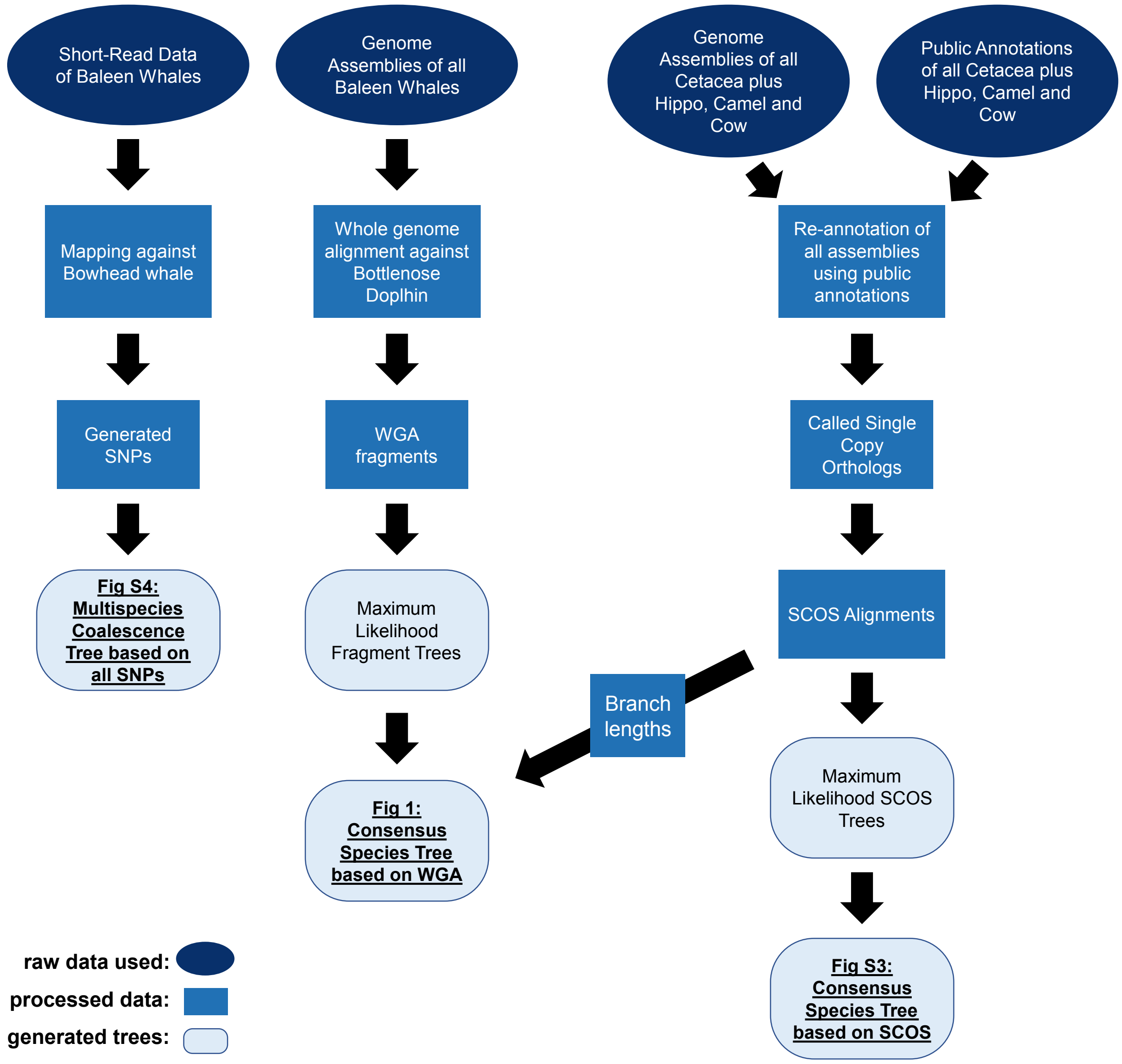

Fig. S6 Pipeline depicting the process of generating all phylogenomic trees. It is differentiated between used raw input data (dark blue circles), processed intermediate data (blue rectangles) and resulting output trees (light blue rounded squares). Final trees presented in either the main manuscript or the supplement are underlined.

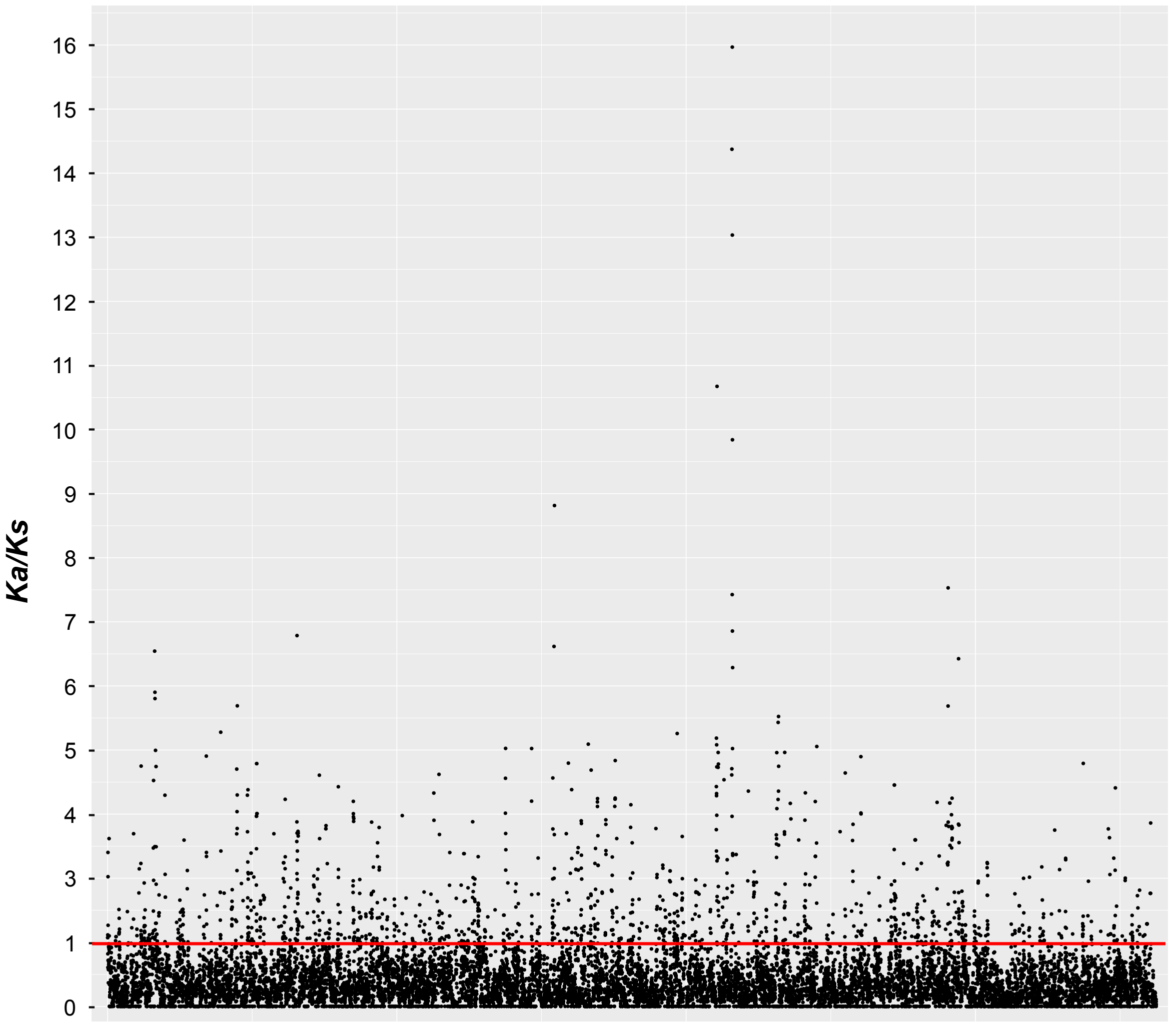

Fig. S7 Distribution of Ka/Ks values over all tested orthologs. Orthologs were inferred between all six baleen whales that resulted from the phylogenetic targeting analysis and the human genome GRCh38. Genes with Ka/Ks > 1 were considered as putative selected (red line) in downstream analyses.

Supplementary Tables

Table S1 Repeat content of the pygmy right whale assembly. Repeats were collected by RepeatMasker v4.1 (www.repeatmasker.org) after modelling and identifying them with RepeatModeler v2 (www.repeatmasker.org) using the Cetartiodactyla database from RepBase (Jurka et al. 2005).

	Number of elements	Length occupied (bp)	Percentage of sequence (%)
Retroelements	2413632	865034774	34.39
**SINEs:**	690495	129626373	5.15
Penelope	66	12853	0.00
**LINEs:**	1354802	596935095	23.73
CRE/SLACS	0	0	0.00
L2/CR1/Rex	297003	65503588	2.60
R1/LOA/Jockey	0	0	0.00
R2/R4/NeSL	355	92064	0.00
RTE/Bov-B	10495	3115804	0.12
L1/CIN4	1046745	528167155	21.00
**LTR elements:**	368335	138473306	5.51
BEL/Pao	0	0	0.00
Ty1/Copia	286	1064306	0.04
Gypsy/DIRS1	14204	3919658	0.16
Retroviral	343447	130769605	5.20
**DNA transposons:**	369981	79396649	3.16
hobo-Activator	263101	52978695	2.11
Tc1-IS630-Pogo	97913	24838964	0.99
En-Spm	0	0	0.00
MuDR-IS905	0	0	0.00
PiggyBac	899	312728	0.01
Tourist/Harbinger	319	56939	0.00
Other transposons	0	0	0.00

(Mirage,Pelement,Transib)			
**Others:**			
Rolling-circles	1547	388897	0.02
Unclassified:	39979	6637640	0.26
Total interspersed repeats:		951069063	37.81
Small RNA:	325970	77630921	3.09
Satellites:	3700	1918182	0.08
Simple repeats:	8338	1139523	0.05
Low complexity:	0	0	0.00

Table S2 QuIBL results for all triplets resulting from combining rorquals species affecting the placement of the gray whale (excluding the two minke whale species). Tested were 1000 randomly selected WGA fragment trees.

triplet	outgr.	C1	C2	ILS-prop.	ILS+ Intro. prop.	num-trees	BIC (ILS + Intro)	BIC (only ILS)	ΔBIC	total prop. non-ILS
Bmus_Mnov_Bric	Bmus	0	52.736	0.987	0.013	77	-664.726	-605.133	-59.593	0.001
Bmus_Mnov_Bric	Bric	0	0.532	0.604	0.396	50	-497.555	-504.347	6.791	0.020
Bmus_Mnov_Bric	Mnov	0	1.537	0.010	0.990	873	-9325.190	-8838.495	-486.695	0.864
Bphy_Bmus_Bric	Bmus	0	7.415	0.939	0.061	88	-654.727	-649.388	-5.339	0.005
Bphy_Bmus_Bric	Bric	0	0.635	0.313	0.687	50	-513.413	-516.890	3.477	0.034
Bphy_Bmus_Bric	Bphy	0	1.560	0.011	0.989	862	-9208.359	-8723.705	-484.654	0.852
Bphy_Erob_Bric	Bphy	0	0.570	0.214	0.786	320	-3555.136	-3524.173	-30.963	0.251
Bphy_Erob_Bric	Erob	0	0.177	0.598	0.402	342	-3215.928	-3222.379	6.451	0.137
Bphy_Erob_Bric	Bric	0	1.315	0.249	0.751	338	-3845.348	-3797.613	-47.735	0.254
Bphy_Mnov_Bric	Bphy	0	0.706	0.487	0.513	31	-301.791	-307.331	5.541	0.016
Bphy_Mnov_Bric	Mnov	0	5.228	0.883	0.117	49	-380.309	-383.908	3.598	0.006
Bphy_Mnov_Bric	Bric	0	1.655	0.000	1.000	920	-9510.059	-8906.339	-603.720	0.920
Bphy_Erob_Bmus	Bphy	0	0.467	0.329	0.671	332	-3585.732	-3572.468	-13.264	0.223
Bphy_Erob_Bmus	Erob	0	0.835	0.208	0.792	327	-3595.573	-3554.387	-41.186	0.259
Bphy_Erob_Bmus	Bmus	0	0.592	0.108	0.892	341	-3774.736	-3717.975	-56.762	0.304
Bphy_Bmus_Mnov	Bphy	0	0.506	0.177	0.823	29	-275.500	-278.007	2.508	0.024
Bphy_Bmus_Mnov	Mnov	0	0.799	0.609	0.391	41	-408.184	-414.814	6.630	0.016
Bphy_Bmus_Mnov	Bmus	0	1.037	0.000	1.000	930	-9192.187	-8742.306	-449.881	0.930
Bphy_Erob_Mnov	Bphy	0	0.423	0.352	0.648	49	-457.953	-462.624	4.671	0.032
Bphy_Erob_Mnov	Mnov	0	9.184	0.941	0.059	41	-380.572	-377.169	-3.403	0.002
Bphy_Erob_Mnov	Erob	0	0.844	0.001	0.999	910	-8847.166	-8491.310	-355.856	0.909
Erob_Bmus_Bric	Bmus	0	0.270	0.773	0.227	73	-661.116	-669.611	8.495	0.017
Erob_Bmus_Bric	Bric	0	0.278	0.238	0.762	74	-678.588	-681.750	3.161	0.056
Erob_Bmus_Bric	Erob	0	1.755	0.138	0.862	853	-8783.866	-8510.811	-273.055	0.736
Erob_Mnov_Bric	Erob	0	0.264	0.476	0.524	339	-3312.733	-3313.683	0.950	0.178
Erob_Mnov_Bric	Mnov	0	0.471	0.277	0.723	328	-3545.221	-3527.425	-17.796	0.237
Erob_Mnov_Bric	Bric	0	0.557	0.148	0.852	333	-3655.329	-3611.483	-43.847	0.284
Erob_Bmus_Mnov	Erob	0	0.858	0.188	0.812	77	-3620.386	-3573.493	-46.893	0.266
Erob_Bmus_Mnov	Mnov	0	0.772	0.582	0.418	50	-3641.136	-3645.268	4.132	0.141
Erob_Bmus_Mnov	Bmus	0	0.550	0.118	0.882	873	-3660.379	-3612.023	-48.355	0.294

triplet: The three-taxon subset considered. Species abbreviations separated by underscores
outgr.: Species inferred to be the outgroup out of the considered triplet.
C(n): Inferred species tree branch length for (1) the ILS-only model and (2) the ILS+introgression model. The ILS model is forced to be 0, as all lineages must be in the same population.
ILS-prop.: inferred proportion of the trees that account for the ILS-only model
ILS+Intro. prop.: inferred proportion of the trees that account for the ILS+introgression model.
num-trees: number of trees in the considered topology
BIC: Value resulted from a Bayesian information criterion test
BIC (n): raw BIC values for one of both models
ΔBIC: difference in BIC value between the models. ΔBIC < -10 was set as a cutoff to decide between the ILS-only model (ΔBIC > -10) and the ILS+introgression model (ΔBIC < -10)
total prop.: total proportion of trees that support the ILS+introgression model calculated as “ILS+Intro. prop * (num-trees / total trees in sample)”.

Table S3 Calibration points used in the date phylogeny.

Defined Node	Fossil record (species)	Suggested age (in Mya)	Literature
Cetartiodactyla	*Diacodexis ilicis*	57.3 - 72.2	Gingerich 1989
Cetancodonta	*Himalayacetus subathuensis*	52.9 - 58.3	Bajpai and Gingerich 1998
Cetacea	*Mystacodon selenensis*	36.1 - 39.6	Lambert et al. 2017
Mysticeti	*Balaenella brachyrhynus*	21.1 - 26.8	Bisconti 2005
Odontoceti	*Arktocara yakataga*	25.1 - 30.8	Boersma and Pyenson 2016

Table S4 Body mass data used for phylogenetic targeting. We collected estimates for mean body-mass (Kg), mean body-length (m) and longevity (years). Eventually, longevity was excluded from the phylogenetic targeting analysis due to the lack of information available.

Species	Length (m)	Mass (Kg)	Longevity	Literature
Caperea marginata	6	3430	?	Budylenko et al. 1973
Balaena mysticetus	19	80000	200	Georg et al. 1999
Eubalaena australis	14	35000	?	Hamilton et al. 1998, Fortune et al. 2021
Eubalaena glacialis	11	35000	70	Christiansen et al. 2019
Eubalaena japonica	16	60000	?	Lockyer 1976
Balaenoptera bonaerensis	9	6800	?	Konishi 2006
Balaenoptera acutorostrata	8,5	5000	50	Markussen et al. 1992, Horwood 1989
Balaenoptera musculus	23	100000	90	Ruud 1956, Gilpatrick and Perryman, Sears and Perrin 2008
Balaenoptera ricei	12	13000	?	Tershy 1992, Rosel et al. 2021
Eschrichtius robustus	14	35000	77	Rice and Wolman 1971, Swartz 2018
Megaptera novaeangliae	14	40000	95	Chittleborough 1959, Jefferson et al. 2015, Clapham and Mead 1999
Balaenoptera physalus	20	45000	90	Lokyer and Waters 1986, Aguilar and Garcia-Vernet 2018

Table S5 Maximal pairs inferred from the phylogenetic targeting analysis. Pairs were inferred using the PhyloTargeting Webserver (https://phylotargeting.nunn-lab.org/index.html) by providing body length and mass data (Supplement Table S4) as well as the phylogenetic tree depicted in Fig. 1. Tursiops truncates was used as an outgroup. Eventually, we did not use the pair of right whales due to uncertainty in the data as well as having the lowest standardized summed score.

Species 1	Species 2	Raw difference (body mass)	Score (body mass)	Raw difference (body length)	Score (body length)	Sum of branch lengths	No. of branches	Summed score (standard.)
**Cmar**	**Bmys**	**76.57**	**0.793**	**13**	**0.765**	**0.081**	**4**	**1.558**
**Egla**	**Ejap**	**25**	**0.259**	**4.5**	**0.265**	**0.017**	**2**	**0.524**
**Bacu**	**Bphy**	**40**	**0.414**	**11.5**	**0.676**	**0.034**	**6**	**1.091**
**Bric**	**Bmus**	**87**	**0.901**	**11**	**0.647**	**0.019**	**2**	**1.548**

Table S6 Used data featured in this study including assemblies, short read archives and proteomes from other *Cetacea* or *Cetartiodactyla*. Provided are information for the scientific and common name, for the database, the respective ID, source publication or project and usage in this study. A list of aberrations can be found below.

<u>Assemblies</u>

Species	Common Name	Database	ID	Literature/Consortium	Usage
Balaena mysticetus	bowhead whale	Bowhead whale genome resource	-	Keane et al. 2015	WGA,SCOS,SNP reference,cancer analyses reference
Eubalaena australis	Southern right whale	DNA Zoo	-	DNA Zoo	WGA,SCOS
Eubalaena glacialis	North Atlantic right whale	DNA Zoo	-	DNA Zoo	WGA,SCOS
Eubalaena japonica	North Pacific right whale	NCBI	GCA_004363455.1	Zoonomia Consortium	WGA,SCOS

Balaenoptera bonaerensis	Antarctic minke whale	NCBI	GCA_000978805.1	Kishida et al. 2015	WGA,SCOS
Balaenoptera acutorostrata	minke whale	NCBI	GCA_000493695.1	Yim et al. 2014	WGA,SCOS,cancer analyses reference
Balaenoptera musculus	blue whale	CNGBdb	CNA0007254	Yuan et al. 2021	WGA,SCOS,cancer analyses reference
Balaenoptera ricei	rice whale	DNA Zoo	-	DNA Zoo	WGA,SCOS
Eschrichtius robustus	gray whale	NCBI	GCA_004363415.1	Zoonomia Consortium	WGA,SCOS
Megaptera novaeangliae	humpback whale	NCBI	GCA_004329385.1	Tollis et al. 2019	WGA,SCOS,cancer analyses reference
Balaenoptera physalus	fin whale	NCBI	GCA_023338255.1	Wolf et al. 2022	WGA,SCOS
Bos taurus	cattle	NCBI	GCA_002263795.3 ARS-UCD1.3	USDA ARS	SCOS
Camelus dromedarius	Arabian camel	NCBI	GCA_000803125.3 CamDro3	Elbers et al. 2019	SCOS
Hippopotamus amphibius	hippopotamus	NCBI	GCA_023065835.1 ASM2306583v1	Northwestern Polytechnology University	SCOS
Physeter catodon	sperm whale	NCBI	GCA_002837175.2 ASM283717v2	Fan et al. 2018	SCOS
Inia geoffrensis	boutu	NCBI	GCA_004363515.1 IniGeo_v1_BIUU	Broad Institute	SCOS
Tursiops truncatus	bottlenose dolphin	NCBI	GCA_011762595.1	VGP	WGA reference,SCOS

Orcinus orca	killer whale	NCBI	GCA_000331955.2 Oorc_1.1	Foote et al. 2015	SCOS
Delphinapterus leucas	beluga whale	NCBI	GCA_002288925.3 ASM228892v3	Jones et al. 2017	SCOS
Monodon monoceros	narwhal	NCBI	GCA_005125345.1	Westbury et al. 2019	SCOS

<u>SRA</u>

Species	Common Name	Database	ID	Literature/Consortium	Usage
Balaena mysticetus	bowhead whale	Bowhead whale genome resource	-	Keane et al. 2015	SNP,cancer analyses
Eubalaena glacialis	North Atlantic right whale	NCBI	SRR11097130	DNA Zoo	SNP
Balaenoptera acutorostrata	minke whale	NCBI	SRR924087	Yim et al. 2014	SNP,cancer analyses
Megaptera novaeangliae	humpback whale	NCBI	SRP175048	Tollis et al. 2019	SNP,cancer analyses
Balaenoptera physalus	fin whale	NCBI	SRP325690	Wolf et al. 2022	SNP, cancer analyses
Eschrichtius robustus	gray whale	NCBI	SRP108933	Arnason et al. 2018	SNP
Balaenoptera musculus	blue whale	NCBI	SRP108933	Arnason et al. 2018	SNP, cancer analyses

Balaenoptera borealis	sei whale	NCBI	SRP108933	Arnason et al. 2018	SNP

Proteoms

Species	Common Name	database	ID	Literature/Consortium	Usage
Balaena mysticetus	bowhead whale	Bowhead whale genome resource	-	Keane et al. 2015	Cmar annotation,SCOS, cancer analyses
Balaenoptera acutorostrata	minke whale	NCBI	GCF_000493695	Yim et al. 2014	Cmar annotation,SCOS, cancer analyses
Balaenoptera physalus	fin whale	NCBI	GCA_023338255.1	Wolf et al. 2022	Cmar annotation,SCOS, cancer analyses
Bos taurus	cattle	NCBI	GCA_002263795.3 ARS-UCD1.3	USDA ARS	Cmar annotation,SCOS, cancer analyses
Camelus dromedarius	Arabian camel	NCBI	GCA_000803125.3 CamDro3	Elbers et al. 2019	Cmar annotation,SCOS, cancer analyses
Delphinapterus leucas	beluga whale	NCBI	GCA_002288925.3 ASM228892v3	Jones et al. 2017	Cmar annotation,SCOS, cancer analyses
Monodon monoceros	narwhal	NCBI	GCA_005125345.1	Westbury et al. 2019	Cmar annotation,SCOS, cancer analyses
Orcinus orca	killer whale	NCBI	GCA_000331955.2 Oorc_1.1	Foote et al. 2015	Cmar annotation,SCOS, cancer analyses
Physeter catodon	sperm whale	NCBI	GCA_002837175.2 ASM283717v2	Fan et al. 2018	Cmar annotation,SCOS, cancer analyses

Tursiops truncatus	bottlenose dolphin	NCBI	GCA_011762595.1	VGP	Cmar annotation,SCOS, cancer analyses

NCBI: National Center for Biotechnology Information
CNGBdb: China National GeneBank DataBase
WGA: Whole-genome Alignment approach (Fig 1, main manuscript)
SCOS: Single Copy Orthologous Sequence approach (Fig. S2)
SNP: Single Nucleotide Polymorphism approach (Fig. S3)
VGP: Vertebrate Genome Project
Cmar: *Caperea marginata*

Supplementary References

Aguilar A, García-Vernet R. Fin whale: Balaenoptera physalus. In: Würsig BG, Thewissen JGM, Kovacs KM, eds. Encyclopedia of marine mammals. Amsterdam: Academic Press; 2017. p. 368–371.

Årnason Ú, Lammers F, Kumar V, Nilsson MA, Janke A. Whole-genome sequencing of the blue whale and other rorquals finds signatures for introgressive gene flow. Sci Adv. 2018;4(4):eaap9873. doi:10.1126/sciadv.aap9873.

Bajpai S, Gingerich PD. A new Eocene archaeocete (Mammalia, Cetacea) from India and the time of origin of whales. Proc Natl Acad Sci U S A. 1998;95(26):15464–8. doi:10.1073/pnas.95.26.15464.

Bisconti M. SKULL MORPHOLOGY AND PHYLOGENETIC RELATIONSHIPS OF A NEW DIMINUTIVE BALAENID FROM THE LOWER PLIOCENE OF BELGIUM. Palaeontology. 2005;48(4):793–816. doi:10.1111/j.1475-4983.2005.00488.x.

Boersma AT, Pyenson ND. Arktocara yakataga, a new fossil odontocete (Mammalia, Cetacea) from the Oligocene of Alaska and the antiquity of Platanistoidea. PeerJ. 2016;4:e2321. doi:10.7717/peerj.2321.

Budylenko GA, Panfilov BG, Pakhomova AA, Sazhinov EG. New data on pygmy right whales Neobalaena marginata (Gray, 1848). Trudy Atlanticheskii Nauchno-Issledovatel'skii Institut Rybnogo Khozyaistva I Okeanografii. 1973;51:122–32.

Chittleborough RG. Determination of age in the humpback whale, Megaptera nodosa (Bonnaterre). Marine and Freshwater Research. 1959;10(2):125–43.

Christiansen F, Sironi M, Moore MJ, Di Martino M, Ricciardi M, Warick HA, et al. Estimating body mass of free-living whales using aerial photogrammetry and 3D volumetrics. Methods Ecol Evol. 2019;10(12):2034–44. doi:10.1111/2041-210X.13298.

Clapham PJ, Mead JG. Megaptera novaeangliae. Mammalian Species. 1999(604):1. doi:10.2307/3504352.

Elbers JP, Rogers MF, Perelman PL, Proskuryakova AA, Serdyukova NA, Johnson WE, et al. Improving Illumina assemblies with Hi-C and long reads: An example with the North African dromedary. Mol Ecol Resour. 2019;19(4):1015–26. doi:10.1111/1755-0998.13020.

Fan G, Zhang Y, Liu X, Wang J, Sun Z, Sun S, et al. The first chromosome-level genome for a marine mammal as a resource to study ecology and evolution. Mol Ecol Resour. 2019;19(4):944–56. doi:10.1111/1755-0998.13003.

Foote AD, Liu Y, Thomas GWC, Vinař T, Alföldi J, Deng J, et al. Convergent evolution of the genomes of marine mammals. Nat Genet. 2015;47(3):272–5. doi:10.1038/ng.3198.

Fortune SME, Moore MJ, Perryman WL, Trites AW. Body growth of North Atlantic right whales (Eubalaena glacialis) revisited. Marine Mammal Science. 2021;37(2):433–47. doi:10.1111/mms.12753.

George JC, Bada J, Zeh J, Scott L, Brown SE, O'Hara T, Suydam R. Age and growth estimates of bowhead whales (Balaena mysticetus) via aspartic acid racemization. Can. J. Zool. 1999;77(4):571–80. doi:10.1139/z99-015.

Gilpatrick JW, Perryman WL. Geographic variation in external morphology of North Pacific and Southern Hemisphere blue whales (Balaenoptera musculus). Journal of Cetacean Research and Management. 2008;10(1):9–21.

Gingerich PD. New Earliest Wasatchian Mammalian Fauna from the Eocene of Northwestern Wyoming: Composition and Diversity in a Rarely Sampled High-Floodplain Assemblage. 1989.

Hamilton PK, Knowlton AR, Marx MK, Kraus SD. Age structure and longevity in North Atlantic right whales Eubalaena glacialis and their relation to reproduction. Mar. Ecol. Prog. Ser. 1998;171:285–92. doi:10.3354/meps171285.

Horwood J. Biology and exploitation of the minke whale. Boca Raton, Fla.: CRC Press; 1990.

Jefferson TA, Pitman RL, Webber MA, editors. Marine mammals of the world: A comprehensive guide to their identification. 2nd ed. Amsterdam: Academic Press; 2015.

Jones SJM, Taylor GA, Chan S, Warren RL, Hammond SA, Bilobram S, et al. The Genome of the Beluga Whale (Delphinapterus leucas). Genes (Basel) 2017. doi:10.3390/genes8120378.

Jurka J, Kapitonov VV, Pavlicek A, Klonowski P, Kohany O, Walichiewicz J. Repbase Update, a database of eukaryotic repetitive elements. Cytogenet Genome Res. 2005;110(1-4):462–7. doi:10.1159/000084979.

Keane M, Semeiks J, Webb AE, Li YI, Quesada V, Craig T, et al. Insights into the evolution of longevity from the bowhead whale genome. Cell Rep. 2015;10(1):112–22. doi:10.1016/j.celrep.2014.12.008.

Kishida T, Thewissen J, Hayakawa T, Imai H, Agata K. Aquatic adaptation and the evolution of smell and taste in whales. Zoological Lett. 2015;1:9. doi:10.1186/s40851-014-0002-z.

Lambert O, Martínez-Cáceres M, Bianucci G, Di Celma C, Salas-Gismondi R, Steurbaut E, et al. Earliest Mysticete from the Late Eocene of Peru Sheds New Light on the Origin of Baleen Whales. Curr Biol. 2017;27(10):1535-1541.e2. doi:10.1016/j.cub.2017.04.026.

Lockyer C. Body weights of some species of large whales. ICES Journal of Marine Science. 1976;36(3):259–73. doi:10.1093/icesjms/36.3.259.

Lockyer C, Waters T. WEIGHTS AND ANATOMICAL MEASUREMENTS OF NORTHEASTERN ATLANTIC FIN (BALAENOPTERA PHYSALUS, LINNAEUS) AND SEI (B. BOREALIS, LESSON) WHALES. Marine Mammal Science. 1986;2(3):169–85. doi:10.1111/j.1748-7692.1986.tb00039.x.

Markussen NH, Ryg M, Lydersen C. Food consumption of the NE Atlantic minke whale (Balaenoptera acutorostrata) population estimated with a simulation model. ICES Journal of Marine Science. 1992;49(3):317–23. doi:10.1093/icesjms/49.3.317.

Rice DW, Wolman AA. The life history and ecology of the gray whale (Eschrichtius robustus). American Society of Mammalogist; 1971.

Rosel PE, Wilcox LA, Yamada TK, Mullin KD. A new species of baleen whale (Balaenoptera) from the Gulf of Mexico, with a review of its geographic distribution. Marine Mammal Science. 2021;37(2):577–610. doi:10.1111/mms.12776.

Ruud JT. The Blue Whale. Scientific American. 1956;195(6):46–51.

Sears R, Perrin WF. Blue Whale: Balaenoptera musculus. In: Perrin WF, Würsig BG, Thewissen JGM, eds. Encyclopedia of marine mammals. 2nd ed. Amsterdam, Boston, Mass.: Elsevier/Academic Press; 2009. p. 120–124.

Swartz SL. Gray Whale: Eschrichtius robustus. In: Würsig BG, Thewissen JGM, Kovacs KM, eds. Encyclopedia of marine mammals. Amsterdam: Academic Press; 2017. p. 422–428.

Tershy BR. Body Size, Diet, Habitat Use, and Social Behavior of Balaenoptera Whales in the Gulf of California. Journal of Mammalogy. 1992;73(3):477–86. doi:10.2307/1382013.

Tollis M, Robbins J, Webb AE, Kuderna LFK, Caulin AF, Garcia JD, et al. Return to the Sea, Get Huge, Beat Cancer: An Analysis of Cetacean Genomes Including an Assembly for the Humpback Whale (Megaptera novaeangliae). Mol Biol Evol. 2019;36(8):1746–63. doi:10.1093/molbev/msz099.

Westbury MV, Petersen B, Garde E, Heide-Jørgensen MP, Lorenzen ED. Narwhal Genome Reveals Long-Term Low Genetic Diversity despite Current Large Abundance Size. iScience. 2019;15:592–9. doi:10.1016/j.isci.2019.03.023.

Wolf M, Jong M de, Halldórsson SD, Árnason Ú, Janke A. Genomic Impact of Whaling in North Atlantic Fin Whales. Mol Biol Evol 2022. doi:10.1093/molbev/msac094.

Yim H-S, Cho YS, Guang X, Kang SG, Jeong J-Y, Cha S-S, et al. Minke whale genome and aquatic adaptation in cetaceans. Nat Genet. 2014;46(1):88–92. doi:10.1038/ng.2835.

Yuan Y, Zhang Y, Zhang P, Liu C, Wang J, Gao H, et al. Comparative genomics provides insights into the aquatic adaptations of mammals. Proc Natl Acad Sci U S A 2021. doi:10.1073/pnas.2106080118.
